# Evolving genetic landscapes of *Plasmodium falciparum* Kelch13 polymorphisms in Nigeria: a systematic review and meta-analysis

**DOI:** 10.1186/s12936-026-06138-x

**Published:** 2026-09-21

**Authors:** Aaron Onyebuchi Nwana, Emmanuel Afolabi Bakare, Happiness Oluwatosin Ismail, Segun Isaac Oyedeji

**Affiliations:** 1 International Centre for Applied Mathematical Modelling and Data Analytics (ICAMMDA), Federal University Oye-Ekiti, Oye-Ekiti, Ekiti State Nigeria; 2 Department of Animal and Environmental Biology, Molecular Parasitology and Genetics Unit, Federal University Oye-Ekiti, Oye-Ekiti, Ekiti State Nigeria; 3 Department of Mathematics, Federal University Oye-Ekiti, Oye-Ekiti, Ekiti State Nigeria

**Keywords:** *Plasmodium falciparum* kelch-13, Molecular epidemiology, Imported malaria, Nigeria

## Abstract

**Background:**

Malaria incidence has plateaued in recent years, with periodic increases observed in some settings. This trend is driven by multiple factors, including health system disruptions, vector and parasite resistance, and environmental changes. The emergence of partial resistance to artemisinin-based combination therapies (ACTs) has contributed to delayed parasite clearance and, in some regions, treatment failure. Hence, the need to study the polymorphisms in the *Plasmodium falciparum* Kelch13 gene from studies conducted in Nigeria and imported malaria cases from Nigeria reported in other countries.

**Methods:**

The systematic review and meta-analysis employed the population, intervention or test, comparison, and outcome (PICO) and preferred reporting items for systematic reviews and meta-analyses (PRISMA) guidelines to formulate the research questions and report the reviewed articles completely in both the studies in Nigeria and imported *Plasmodium falciparum* kelch-13 (*Pfk13*) studies from Nigeria reported in other countries. The review included studies that contained any kind of mutation in the *Pfk13* gene from *P. falciparum* and excluded reports for mutations of other antimalarial resistance. The reviewed articles were generated from PubMed, Springer Nature, and Google Scholar using the litsearchr and metagear packages in R. Heterogeneity analyses for the articles and reported SNPs were carried out. Furthermore, the ordinary least squares (OLS) network model was used to assess the effect of distance on *Pfk13* SNP occurrence, while the geostatistical model was used to predict the frequency of *Pfk13* SNP in the country.

**Results:**

In Nigeria, about 124 different *Pfk13* single nucleotide polymorphisms (SNPs) have been reported, which are predominantly non-synonymous mutants domiciled in the kelch-13 propeller domain. The studies were heterogeneous in reporting SNPs occurrence ($I^2$ = 95.3%, $\tau ^2$ = 0.0683, p< 0.0001), and no dominant SNP was reported from the pooled studies ($I^2$ = 36.0%, $\tau ^2<$ 0.0001, p< 0.0001). The network model showed no correlation between the spatial locations of the states and the similarity of their *Pfk13* alleles (β = 0.00035, *p* = 0.51), with high nucleotide diversity (π = 2.31) of the *Pfk13* SNPs. A total of three validated (C580Y, F446I, and A675V) and one candidate (R515K) *Pfk13* SNPs were present from the various reports, and the predicted *Pfk13* mutation frequency showed a marginally higher occurrence in the Southeast and South-South regions of the country and Lagos State.

****Conclusion**:**

Highly diverse and independent emergence of *Pfk13* SNPs together with those that are responsible for artemisinin partial resistance has been reported in Nigeria. These patterns underscore the urgent need for sustained *Pfk13* genomic surveillance and ACT therapeutic efficacy studies to preempt and mitigate the establishment and spread of artemisinin- and partner drug-resistant strains in the country.

**Supplementary Information:**

The online version contains supplementary material available at https://doi.org/10.1186/s12936-026-06138-x.

## Introduction

Malaria remains a major public health challenge in Nigeria, which bears one of the highest malaria burdens globally and accounts for a substantial proportion of malaria cases and deaths in sub-Saharan Africa [1]. The majority of infections in the country are caused by *Plasmodium falciparum*, for which artemisinin-based combination therapies (ACTs) remain the cornerstone of treatment [1, 2]. However, the emergence and spread of antimalarial drug resistance threaten the effectiveness of these therapies and could undermine progress in malaria control.

Artemisinin resistance is characterized by delayed parasite clearance following treatment and is strongly associated with nonsynonymous mutations in the *P. falciparum* kelch 13 (*PfK13*) gene [3]. Although the best-characterized artemisinin resistance-associated mutations initially emerged in the Greater Mekong Subregion, validated and candidate *PfK13* mutations have increasingly been reported in African parasite populations [4, 5]. The detection of these polymorphisms in Africa is of particular concern because they may reflect the emergence, spread, or introduction of parasite lineages with reduced artemisinin susceptibility. Importantly, not all *PfK13* polymorphisms are associated with resistance [6], making characterization of their prevalence, diversity, and geographic distribution essential for interpreting their potential epidemiological and therapeutic significance.

In Nigeria, *PfK13* polymorphisms have been reported in parasite populations from several states, with studies documenting considerable variation in the mutations detected [7]. However, the available evidence remains geographically fragmented and varies in study design and sampling locations. Consequently, the national distribution and diversity of reported *PfK13* single nucleotide polymorphisms (SNPs), as well as their potential implications for artemisinin resistance surveillance, remain poorly synthesized. A comprehensive assessment of the available Nigerian evidence is therefore needed to identify geographic patterns, mutation frequency, and gaps in current surveillance.

This review aims to synthesize published evidence on *PfK13* SNPs reported among *P. falciparum* isolates from Nigeria, including evidence from Nigerian patients diagnosed with imported malaria outside the country. Specifically, the study examine the prevalence, diversity, and geographic distribution of reported *PfK13* polymorphisms and consider their potential relevance to artemisinin resistance surveillance in Nigeria.

### Molecular surveillance of antimalarial drug resistance: advances and implications

Before significant advances in bioinformatics and molecular diagnostic tools, the detection of *P. falciparum* resistance to antimalarial drugs relied heavily on *in vivo* and *in vitro* drug sensitivity studies and therapeutic efficacy studies (TES) [8]. Although valuable, TES are time-consuming and resource-intensive and often detect resistance only after it has become clinically significant and widespread [9]. This limitation contributed to the delay in recognition and response to resistance in *P. falciparum* against widely used antimalarials such as chloroquine (CQ) and sulfadoxine-pyrimethamine (SP), allowing resistance to become established in many endemic regions before effective policy changes could be implemented [10].

In contrast, recent developments in molecular surveillance of resistance markers have enabled earlier detection of emerging resistance by identifying specific genetic mutations associated with drug resistance [11]. These molecular markers offer predictive insights that allow for proactive adjustments in treatment policies and drug development strategies [12]. In particular, several haplotypes and single nucleotide polymorphisms (SNPs) in key parasite genes have been validated as reliable indicators of antimalarial resistance [13].

For example, the SVMNT and CVIET haplotypes (wild type: CVMNK) at codons 72 to 76 of the *Pfcrt* gene are associated with resistance to CQ and amodiaquine [14, 15]. Also, mutation in position C350R of *Pfcrt* confers protection against piperaquine [14]. SNPs in the *Pfmdr-1* gene, such as N86Y, Y184F, S1034C, and N1042D, have been implicated in modulating sensitivity to multiple drugs, including CQ, amodiaquine, lumefantrine, and mefloquine [14]. Similarly, resistance to SP has been linked to point mutations in the *Pfdhps* gene (notably, K437G, K540E, A581G, and A613S/T, S436A) and the *Pfdhfr* gene (N51I, C59R, S108N/T, and I164L), which affect the efficacy of sulfadoxine and pyrimethamine, respectively [14].

Furthermore, A16V and S108T mutants of the *Pfdhfr* gene confer resistance to proguanil, and mutation in *Pfcytb* at codon Y268N/S/C has been validated to cause resistance to atovaquone [14]. While multiple copies of the plasmepsin 2/3 (*Pfpm2/3*) genes have been associated with piperaquine resistance [14]. More recently, mutations in the *PfK13* gene have emerged as primary markers of resistance to artemisinin derivatives (artesunate, artemether, and dihydroartemisinin), marking a critical turning point in the global effort to monitor and contain drug-resistant malaria [16–18]. Collectively, these molecular markers form the foundation of modern antimalarial resistance surveillance programs and have become indispensable in guiding national malaria control policies and informing WHO malaria treatment guidelines, whether in chemoprevention or case management [19, 20].

### ACT treatment and treatment effectiveness

The World Health Organization recommends that children and adults with non-severe malaria cases should be treated with a three-day regimen of any of the recommended ACTs: artemether-lumefantrine (AL), artesunate-amodiaquine (ASAQ), artesunate-mefloquine (ASMQ), dihydro-artemisinin-piperaquine (DHAP), artesunate-sulfadoxine-pyrimethamine (ASSP), and the newly recommended artesunate-pyronaridine (ASPY). [20]. The last two are not recommended for pregnant women in their first trimester, while ASPY is not to be used by individuals with liver problems [20]. The artemisinin (ART) derivatives and partner drugs as used in ACTs specifically target the asexual blood-stage (ABS) parasites and, by extension, the early sexual forms. Therefore, the parasites’ ABS resistance to ACTs can also prevent gametocyte sensitivity to ACTs [14]. Primaquine has been investigated to act as a transmission blocking agent, acting against the sexual stages of malaria parasites with no known effect on ABS [21]. Therefore, WHO recommends a single dose of primaquine together with ACTs except during pregnancies in low transmission settings that have no risk of G6PD toxicity to prevent transmissibility of treated malaria [19, 20]. In this light, primaquine offers a low risk of resistance development due to its gametocytocidal ability, therefore preventing the transmission of drug-resistant parasites [14, 21].

Quinine has been previously recommended for pregnant women in their first trimester [19]. However, due to its poor clinical outcome and the long treatment plan of seven days, AL is now recommended in the first trimester of pregnancies [20]. On the other hand, WHO recommends that adults and children of all categories with severe malaria should be treated with intravenous or intramuscular artesunate for at least one day, and this should be sustained until they can tolerate oral medication, after which patients can be treated as in the case of uncomplicated malaria [19, 20].

With continuous use of ACTs as the first-line treatment and consideration for its use for mass drug administration (MDA) as part of supplementary intervention, the common African variants of *Pfk13* mutations, such as R561H and A675V [5, 22–24] could be selected to become dominant due to selective drug pressure [25]. Their presence and spread across Africa will severely impede the efficacy of artemisinin-based treatments and could potentially undermine the progress made thus far in eliminating malaria on the continent.

In this light, ACTs used for malaria treatment in a country’s national malaria policy are not recommended for other drug-based malaria interventions such as IPTp, perennial malaria chemoprevention (PMC), SMC, intermittent preventive treatment in school children (IPTsc), or MDA. Nigeria adopted the use of ACTs with AL as the first line of treatment and ASAQ and DHP on some occasions [26], while ACTs such as ASSP are not recommended owing to the high resistance to SP in the country [27, 28]. Unlike countries in Southeast Asia that had to change their first-line ACTs, Nigeria has continued to use AL since its introduction in 2005 [26]. According to the WHO, a country should consider changing an antimalarial medicine recommended in the national malaria treatment policy if the total proportion of treatment failure observed during therapeutic efficacy studies (TES) is ≥ 10% [3]. However, AL remains still effective in Nigeria, as TES studies conducted in the country still show >95% of the effectiveness of AL, ASAQ, and DHAP [27].

Drug resistance has been defined as the ability of the parasite to survive and/or multiply despite the administration and absorption of a drug given in doses equal to or higher than those usually recommended but within the tolerance of the subject [3]; with respect to artemisinin resistance, a partial resistance is known to occur, resulting in delayed parasite clearance following treatment with artemisinin monotherapy or with an artemisinin-based combination therapy (ACT) [3]. In addition, the concomitant emergence of partner drug resistance is now causing high ACT treatment failure rates in several areas [3, 14, 29]. Antimalarial resistance can arise through two main mechanisms. First, resistance may result from changes in drug accumulation, such as reduced uptake or increased efflux of the drug by the parasite, as seen mostly with drugs such as chloroquine, amodiaquine, quinine, halofantrine, and mefloquine [14]. Secondly, resistance may arise from mutations in the genes encoding the drug’s target, decreasing the binding affinity of the drug [14]. This mechanism underlies resistance to pyrimethamine, cycloguanil, sulfonamides, atovaquone, and artemisinin [14].

The use of molecular resistance markers to identify parasite-resistant strains has allowed not only the identification of resistant parasite strains but also the prediction of how quickly a resistant strain emerges and spreads [15]. Traditional identification of resistant parasite strains is by in vivo and/or in vitro drug susceptibility assays. Although these methods are effective in identifying resistant strains, they face a host of challenges, the most profound challenge being the cost and time associated with them [15].

### Emergence and spread of antimalarial drug resistance: historical perspective and current threats

*P. falciparum* generally employs the mechanism of genetic recombination and nucleotide substitution to survive pressures from the host immune system and antimalarial drugs [30]. This is evidenced in low-transmission regions like Asia and South America, where malaria parasites exhibit low genetic diversity and small population sizes, therefore limiting recombination and accelerating fixation of resistant alleles [31]. However, in high-transmission areas like Africa, malaria parasites have frequent polyclonal infections, greater genetic recombination, and higher host immunity, creating conditions that favour the persistence and resurgence of drug-sensitive malaria parasite strains [31].

Chloroquine (CQ) and sulfadoxine-pyrimethamine (SP) were once the cornerstone therapies for the treatment of uncomplicated malaria. However, due to widespread reports of treatment failure and confirmed resistance, both drugs were officially withdrawn as first-line treatments in Nigeria [26]. This shift was prompted by the well-documented spread of CQ and SP resistance from Southeast Asia (SEA) to sub-Saharan Africa [32]. The emergence and subsequent dissemination of resistant parasite strains significantly undermined the efficacy of these monotherapies, necessitating the global transition to ACTs as the recommended treatment for uncomplicated malaria [33].

In a concerning parallel to historical patterns, partial resistance to artemisinin is now firmly established in SEA, driven predominantly by SNPs in the *PfK13* gene [16]. Validated SNPs such as C580Y, R539T, I543T, Y493H, F446I, N458Y, M476I, P553L, R561H, C469Y, P574L, A675V, and R622I have been shown to confer delayed parasite clearance following ACT treatment and have been designated as markers of artemisinin partial resistance [3, 18, 34], while SNPs such as P441L, G449A, C469F, A481V/C, R515K, P527H, G538V, N537I/D, and V568G are known to be candidate SNPs for artemisinin partial resistance [3, 18, 34] and D452E, K479I, R515K, S522C, N537D, R575K, M579I, D584V, P667T, and H719N as potential markers [35].

A growing concern is the recent detection of these *PfK13* resistance-associated mutations in Africa, raising fears of a continental spread similar to that previously observed for CQ and SP resistance. For example, R561H and A675V have been independently reported in Uganda [5, 36], suggesting local emergence rather than importation. In Kenya, studies have documented the presence of A675V, C469Y, V568G, P574L, P553L, and R561H mutations, although at low frequencies [22, 37, 38]. In Ethiopia, the research by [37, 39] identified the candidate SNP R622I circulating among field isolates. Furthermore, R561H and A675V have been reported in Rwanda [23, 24]; R561H, R622I, and A675V in Tanzania [40, 41]; other validated mutations, such as A481C and P553L, were reported in Ghana and Angola, respectively [42, 43], and then C580Y was reported in imported cases of travelers from Ghana to China [44].

Although the phenotypic consequences of several African variants of *PfK13* mutations, such as A578S, which is widespread [34], remain unclear. These findings underscore the urgent need for molecular surveillance, timely resistance mapping, and robust containment strategies. The continued efficacy of ACTs in Africa is based on early detection and response to resistance signals before widespread clinical failure occurs.

### The Pf-kelch13 gene

The *P. falciparum* Kelch13 (*PfKelch13*) protein, encoded by the *PfK13* gene (PF3D7-1343700), is located on chromosome 13 of the parasite and encodes a 726-amino-acid protein comprising distinct structural domains [17]. At the N-terminus lies a coiled-coil domain (CCD), followed by an intervening region (IVR) that connects to the broad-complex, tramtrack, and bric-à-brac (BTB)/poxvirus and zinc finger (POZ) domain [17, 45]. The C-terminal portion contains the Kelch domain, also referred to as the Kelch-repeat propeller (KRP) domain, which consists of six β-propeller blades [7, 17, 45]. Notably, nearly all *PfKelch13* allelic variants associated with artemisinin resistance are localized within the KRP domain, highlighting its critical role in drug susceptibility [14]. This domain facilitates protein–protein interactions, often acting as a scaffold for complex formation or regulatory functions [46]. In *P. falciparum*, *Kelch13* is involved in protein ubiquitination and intracellular trafficking, which could affect parasite stress response mechanisms, protein turnover, and drug resistance pathways [46].

### Imported malaria cases

Imported malaria cases remain a global concern towards malaria elimination [47]. This refers to cases of malaria infections acquired outside the area in which it was diagnosed [48]. For countries that are approaching or have achieved elimination, imported malaria presents a high risk for resurgence or re-establishment due to the massive influx of migrants from endemic countries into non-endemic areas throughout the world [47, 49]. For example, from 2012 to 2018, 20,938 cases of imported *P. falciparum* malaria was recorded in China [44] with the African continent contributing the majority of the cases [48, 50, 51]. Active molecular surveillance for imported drug-resistant malaria parasites is a key activity to provide adequate information on the spread of resistant parasites and prepare adequate treatment guidelines [48]. It has been recommended that clinicians in non-endemic regions maintain a high index of suspicion of malaria in febrile patients with recent travel to endemic areas, and when available, rapid diagnostic tests should be utilized alongside microscopy to improve diagnostic precision, as prompt initiation of guideline-directed treatment is essential, as delays in therapy may result in adverse clinical outcomes [52]. Currently, monitoring molecular markers in imported malaria cases provides added support to screen for emerging drug resistance in countries where malaria is endemic, especially considering the cost of such molecular analysis in low- and middle-income countries, particularly in those where field surveillance is challenging. This review also looks at *Pfk13* mutations in imported malaria cases from Nigeria reported in other countries [53]. This will determine whether validated SNPs have been reported in those imported malaria cases from Nigeria as a major contributor to imported malaria cases in various countries, particularly China, this will strenthen the survillance study of artemisining resistance in the country.

## Materials and methods

### Data source

A systematic literature review was conducted using the Preferred Reporting Items for Systematic Reviews and Meta-Analyses (PRISMA) guideline to identify studies on *PfK13* gene mutations in Nigeria. Three databases, PubMed, Springer Nature, and Google Scholar, were searched using a set of naive search words: “Artemisinin, *PfK13*, and Nigeria”. Using the naive search terms in PubMed, a new Boolean search term was generated using the “litsearchr” package in R software. The search term was then supplied to PubMed to generate relevant publications, while the naive search term was used to generate publications from Springer Nature and Google Scholar (Supplementary Table 1).

### Eligibility criteria

**Inclusion criteria**

The systematic review and meta-analysis at the report screening stage included studies that contained any kind of mutation (synonymous and non-synonymous) in the *Pfk13* gene from *P. falciparum* malaria cases in Nigeria or imported *P. falciparum* malaria cases from Nigeria reported in other countries.

**Exclusion criteria**

The exclusion criteria were studies with no *PfK13* mutations, review studies, reports for other countries, mutations of other antimalarial resistance (*Pfcrt, Pfmdr, Pfdhfr, Pfdhps, Pfcytb, Pfatp6, Pfcg2*) molecular markers, and theses already published. The reviewed articles and the data sourced from those papers can be obtained in the supplementary material, Tables S2 and S3.

### Review process

The research articles identified from searches of the electronic databases were screened for eligibility based on their titles and abstracts using the “metagear” package in R. Articles that were not eligible and duplicates were removed based on the inclusion and exclusion criteria stated earliar. The full articles of the selected studies were read to confirm that they all met the inclusion criteria before the data were extracted.

### Data items

The selection and inclusion of the studies was carried out according to the PICOS framework. The framework includes:

**Population (P)**: Reports from studies in Nigeria of individuals confirmed with *P. falciparum* infection, from whom parasite DNA was obtained for the genotyping of the *PfK13* gene.

**Intervention/Exposure/Test (I/E/T)**: Presence of one or more *PfK13* SNPs in the included studies.

**Comparison (C)**: Validated *PfK13* and other non-validated mutant *PfK13* SNPs. Stratification of *PfK13* SNPs by location, imported *PfK13* SNPs, synonymous and non-synonymous *Pfk13* mutations, and mutations within and outside the kelch13 propeller domain.

**Outcome (O)**: Measures derived from this systematic review included the prevalence of *PfK13* mutations, the prevalence of specific *PfK13* SNPs, the spatial distribution of *PfK13* SNPs, and nucleotide diversity.

### Analysis

Descriptive statistics were used to show the number and type of mutations present in various studies in Nigeria and imported *PfK13* SNPs from Nigeria reported in other countries. The chi-square test was used to assess the significance of various attributes such as synonymous and non-synonymous *Pfk13* mutations and SNPs within and outside the kech13 propeller domain. Furthermore, a heterogeneity test was carried out for all included studies and also for individual *PfK13* mutations from each study. In addition, the ordinary least squares (OLS) network model was used to assess the interconnection between mutations and geographic locations. Individual studies carried out in Nigeria were presented on a point map based on the locations where such studies were conducted. Then point mutations for the states were merged so that the prevalence of the *Pfk13* mutation for each state was obtained from the reports in the studies. Furthermore, a geostatistical model was carried out using R-INLA [54], to predict the frequency of *PfK13* mutations in states where no such report existed with the use of 2021 malaria prevalence data from the Nigeria Malaria Indicator Survey [55] and the 2021 data of non-ACT usage for the treatment of uncomplicated malaria from the National Malaria Data Repository (not publicly available) as covariates. All analyses were performed using R software.

## Results

### Study identification, screening, and inclusion process

**Fig. 1:**
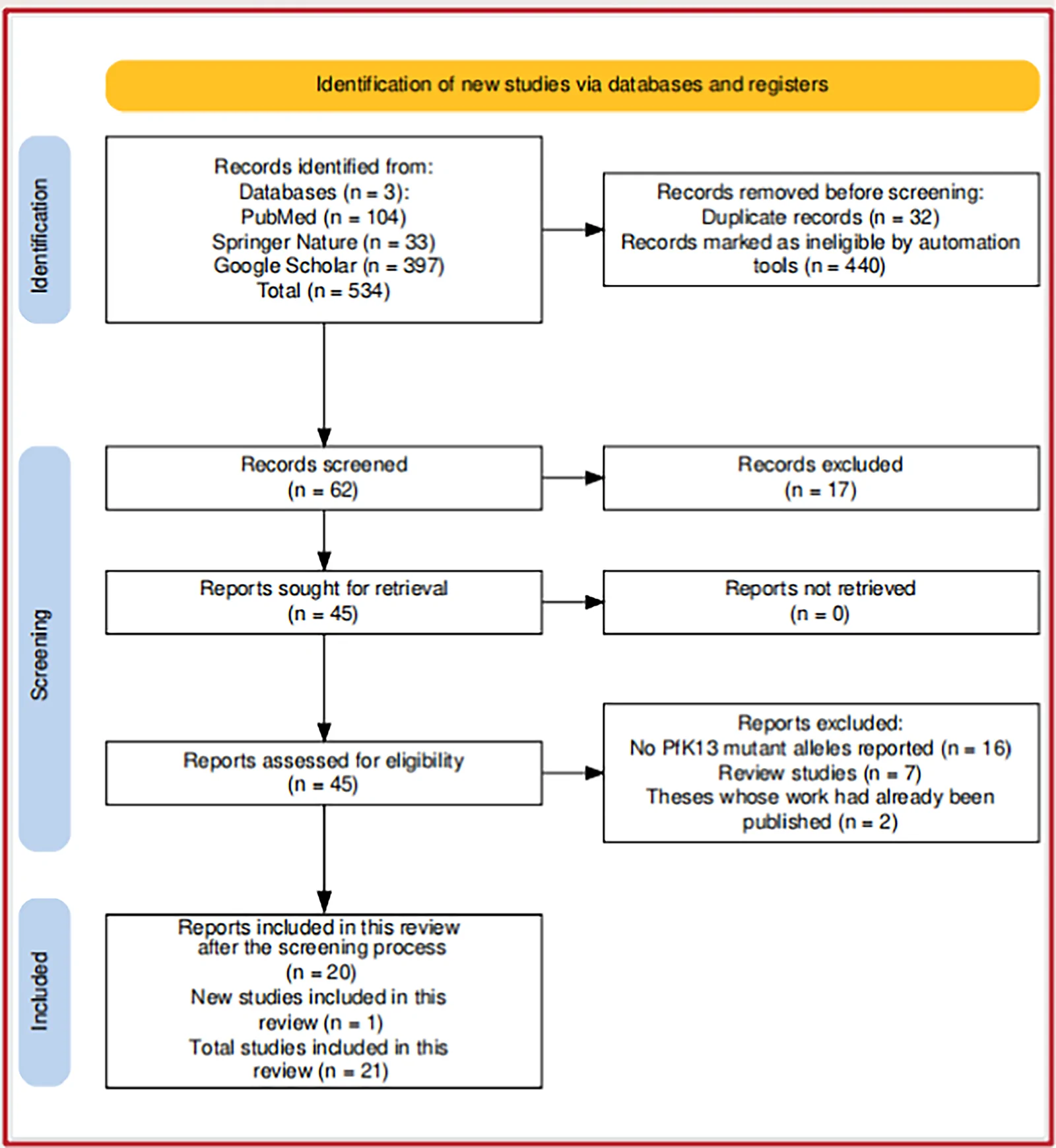
PRISMA flow chart based on [57] for studies included in the systematic review and meta-analysis on *PfK13* mutant alleles from studies conducted in Nigeria (Table S2) and imported malaria cases from Nigeria reported in other countries (Table S3). A recent study by [56], which was published after the entire selection process of this review was included

The database search identified a total of 534 published articles based on the search terms (Fig. 1). Duplicates (n=32) and articles that were considered ineligible (n=440) during the title and abstract screening process were removed. A total of 62 studies were then further screened for inclusion. During this process, 17 studies were removed, leaving 45 studies, which were all retrieved. The retrieved reports were further screened for eligibility, during which 25 reports were removed. The studies that were finally included in the review are 20, comprising nine studies in Nigeria (Fig. 5) and eleven studies of imported malaria cases from Nigeria that were reported in other countries (Fig. 6). Furthermore, a recently published study was added [56], making a total of 21 studies included in the review. The majority of the identified *PfK13* SNPs in the country were nonsynonymous mutations (136 of 168; 80.95%; $\chi ^2$ = 64.38, p< 0.0001). Similarly, most of the detected SNPs were located within the propeller domain (140 of 168; 83.33%; $\chi ^2$ = 74.67, p< 0.0001) (Table S2).

### Frequency of *PfK13* SNPs in studies conducted in Nigeria and imported malaria cases from Nigeria reported in other countries

**Fig. 2:**
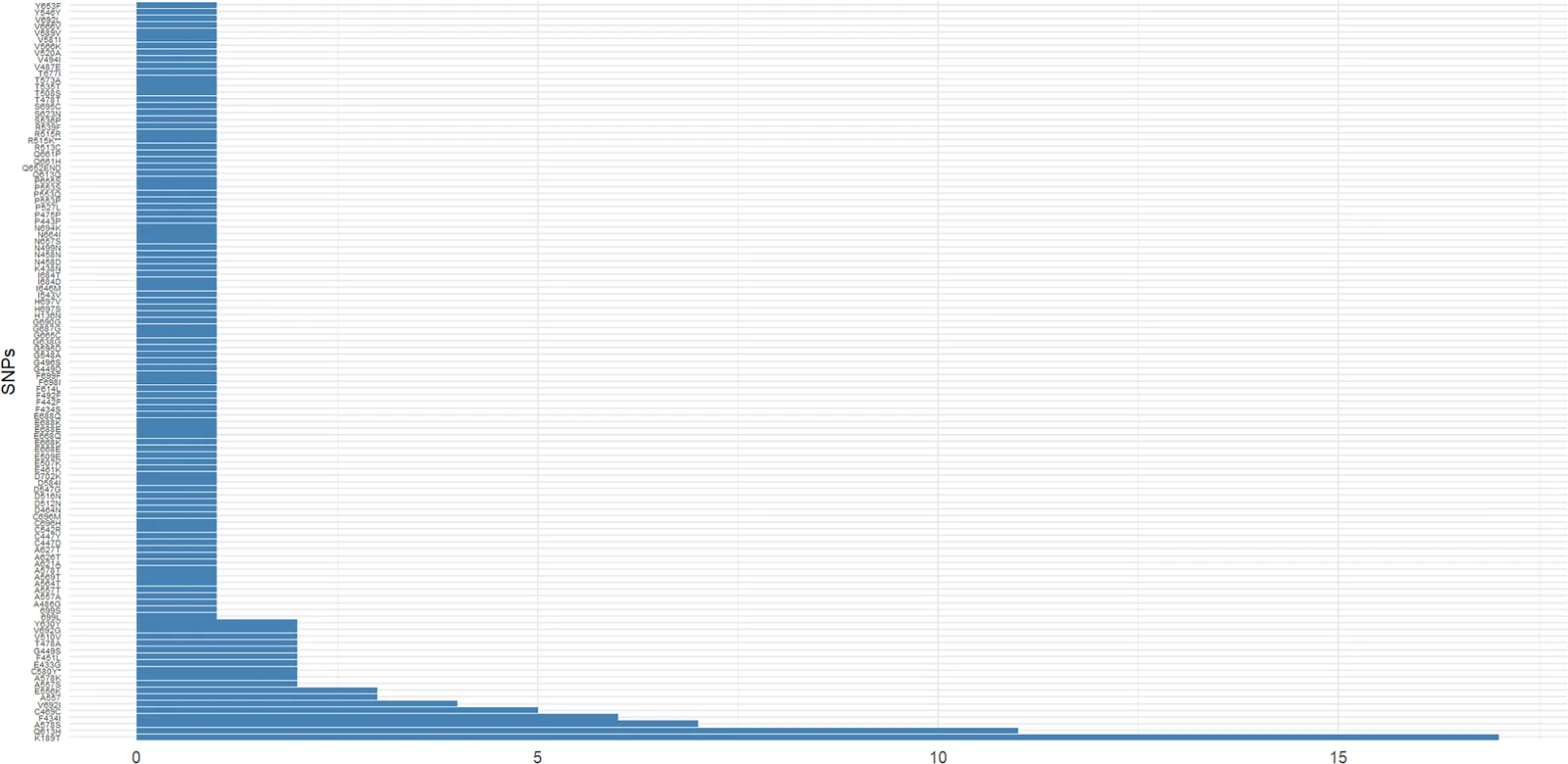
Frequency of *PfK13* gene SNPs in Nigeria. $$^{*}$$ WHO-validated SNPs. $$^{**}$$ WHO candidate SNPs

**Fig. 3:**
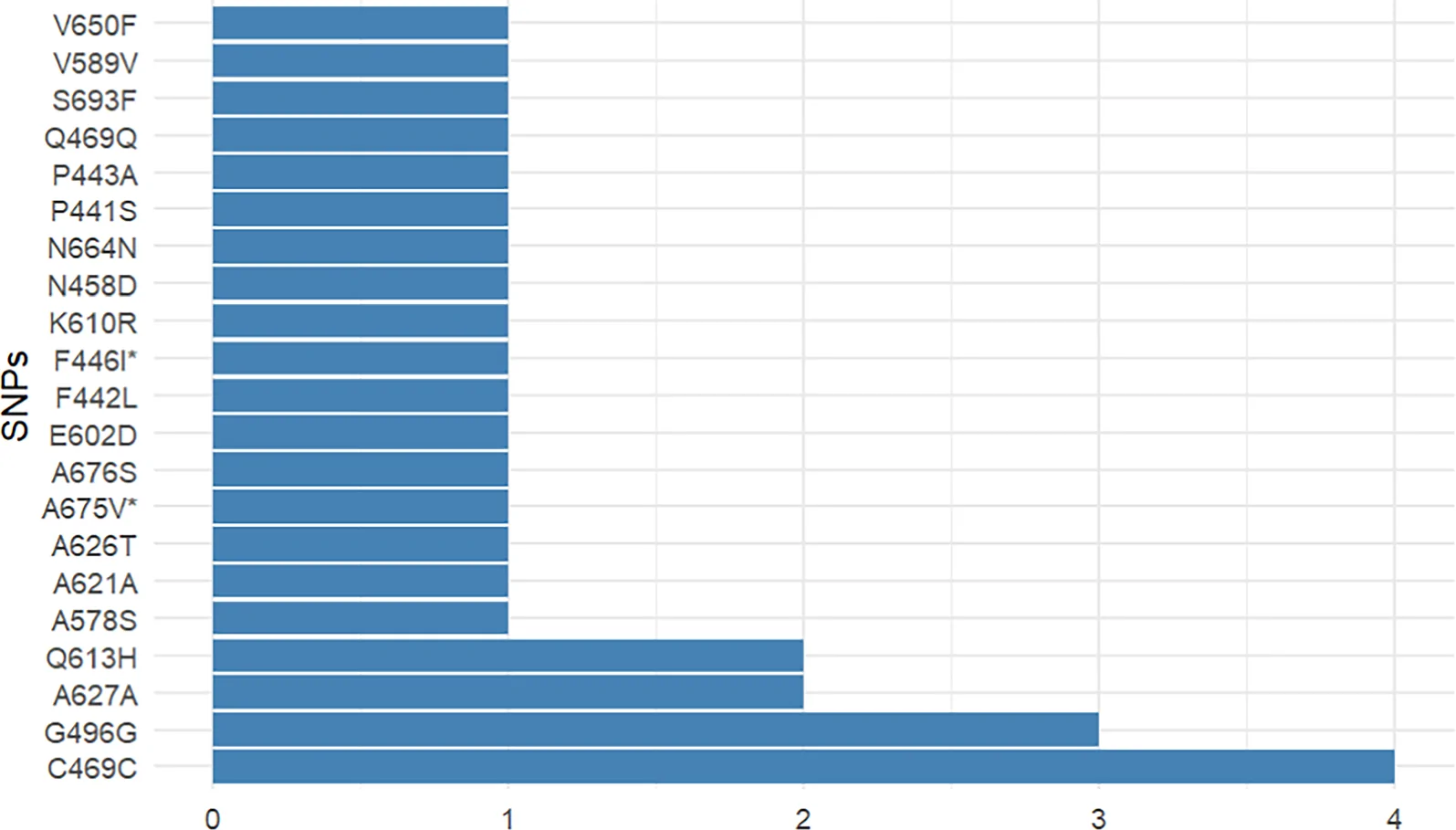
Frequency of *PfK13* SNPs in imported malaria studies from Nigeria reported in other countries. $^{*}$ WHO-validated SNPs

In this review, a total of 110 unique *PfK13* SNPs were identified from studies in Nigeria (Fig. 2), while a total of 21 unique*PfK13* SNPs were reported from studies of imported malaria cases from Nigeria detected in other countries (Fig. 3). In total, 124 unique *PfK13* SNPs were reported, after subtracting duplicate SNPs. A578S, Q613H, N458D, A626T, V589V, C469C, and A621A are common between the Nigerian studies and imported malaria cases from Nigeria reported in other countries. The most SNPs reported across states were Q613H, followed by A578S, and then F434I. K189T was reported with high frequency only in a single study. Furthermore, all the studies used Sanger sequencing approach for determining *PfK13* mutations except for [58]; while [59] used both the Sanger sequencing and next-generation sequencing (NGS) approaches (Table S4).

### Temporal trends in reported *PfK13* polymorphisms

**Fig. 4:**
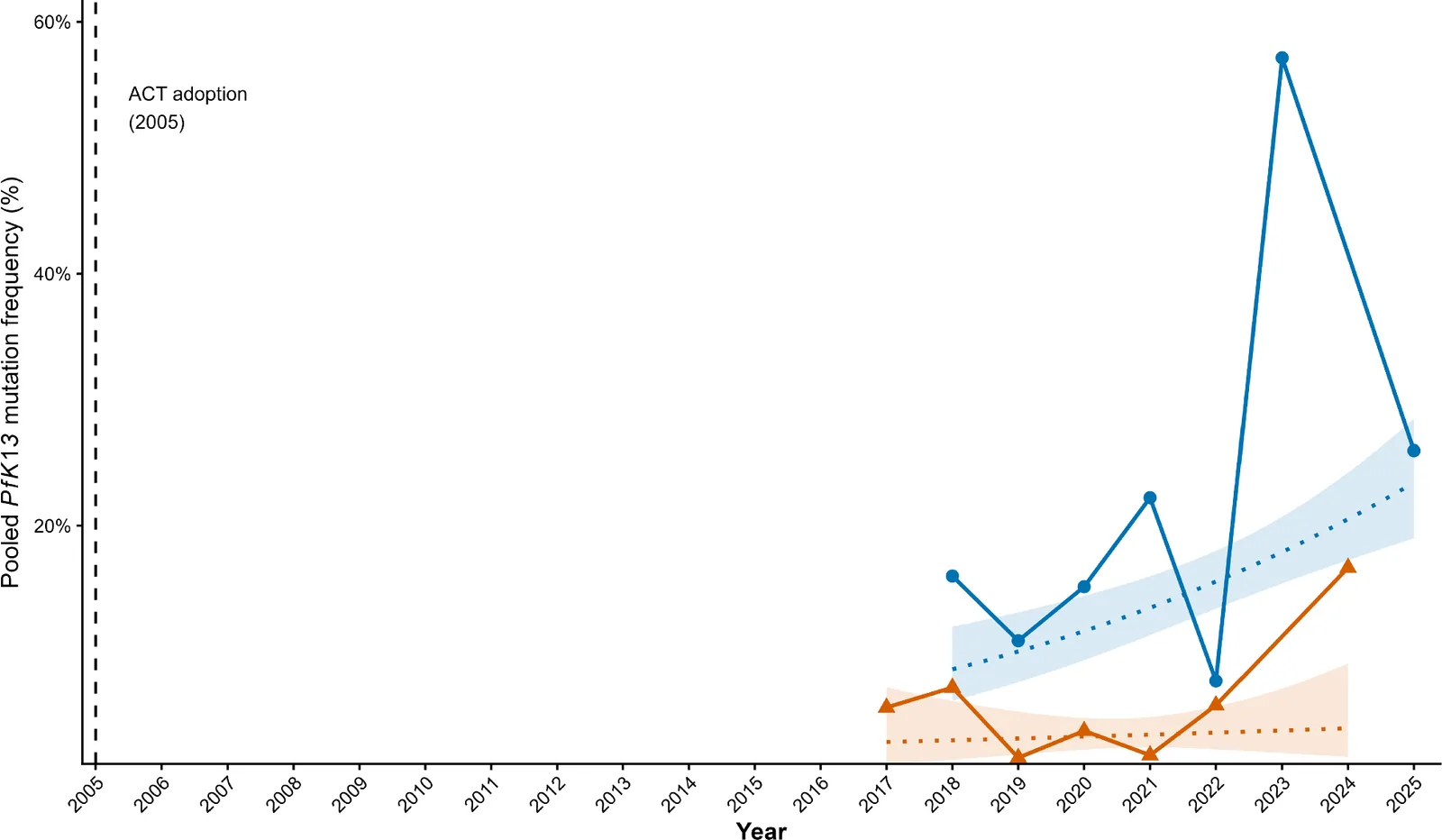
Temporal trends in reported *PfK13* mutation frequency among studies conducted in Nigeria and imported malaria cases originating from Nigeria, 2017–2025. Dotted lines represent fitted temporal trends from binomial logistic regression, with shaded bands indicating the corresponding 95% CIs. The vertical dashed line indicates the introduction of ACT as first-line treatment in Nigeria in 2005. No *PfK13* observations were available before 2017; therefore, the fitted trends are not extrapolated to the pre-observation period

Across the 21 studies included in the temporal analysis (Fig. 4), 187 *PfK13* mutation events were identified among 1,948 parasite isolates from the reviwed articles (Table 1). The overall pooled frequency of reported *PfK13* polymorphisms was higher among studies conducted in Nigeria (155/990; 15.7%) than among imported cases originating from Nigeria and reported outside the country (32/958; 3.3%). The annual pooled frequency varied substantially over time, with frequencies in Nigerian studies ranging from 7.7% in 2022 to 57.1% in 2023, while frequencies among imported cases ranged from 1.6% in 2019 to 16.7% in 2024.

Binomial regression demonstrated a significant overall temporal increase in the odds of detecting a reported *PfK13* polymorphism (OR = 1.30 per year, 95% CI 1.21–1.41, P<0.001). When stratified by study source, this increasing trend remained significant among studies conducted in Nigeria (OR = 1.18 per year, 95% CI 1.10–1.28, P<0.001), but was not significant among imported cases from Nigeria (OR = 1.05, 95% CI 0.83–1.37, P=0.703). After accounting for study source, year remained a significant predictor of reported mutation frequency (P<0.001). However, the year-by-source interaction was not significant (P=0.364), indicating insufficient evidence that the temporal slope differed significantly between the two study groups.

**Table 1:** Temporal distribution and regression analysis of reported *PfK13* polymorphisms, 2017–2025

Study group/year	Studies	Events	N	Frequency (%)
Studies conducted in Nigeria				
2018	1	4	25	16.0
2019	2	24	221	10.9
2020	2	20	132	15.2
2021	1	8	36	22.2
2022	2	23	300	7.7
2023	1	8	14	57.1
2025	1	68	262	26.0
Overall	10	155	990	15.**7**
Imported cases originating from Nigeria				
2017	1	4	72	5.6
2018	1	2	28	7.1
2019	2	2	128	1.6
2020	1	1	27	3.7
2021	3	8	453	1.8
2022	2	14	244	5.7
2024	1	1	6	16.7
Overall	11	32	958	3.3
Temporal regression analysis				
Overall temporal trend				OR = 1.30 (95% CI 1.21–1.41), P<0.001
Nigeria studies				OR = 1.18 (95% CI 1.10–1.28), P<0.001
Imported cases				OR = 1.05 (95% CI 0.83–1.37), P=0.703
Year effect after adjusting for source				P<0.001
Year × source interaction				P=0.364

Frequency represents the pooled proportion of isolates with reported *PfK13* polymorphisms within each year and study group. OR, odds ratio; CI confidence interval. Temporal trends were assessed using binomial logistic regression with the number of mutation events and the number of non-events as the response. An OR >1 indicates increasing odds of detecting a reported *PfK13* polymorphism per calendar year. The year × source interaction tested whether temporal trends differed between Nigerian studies and imported cases

### Heterogeneity of included studies and reported *PfK13* SNPs

**Fig. 5:**
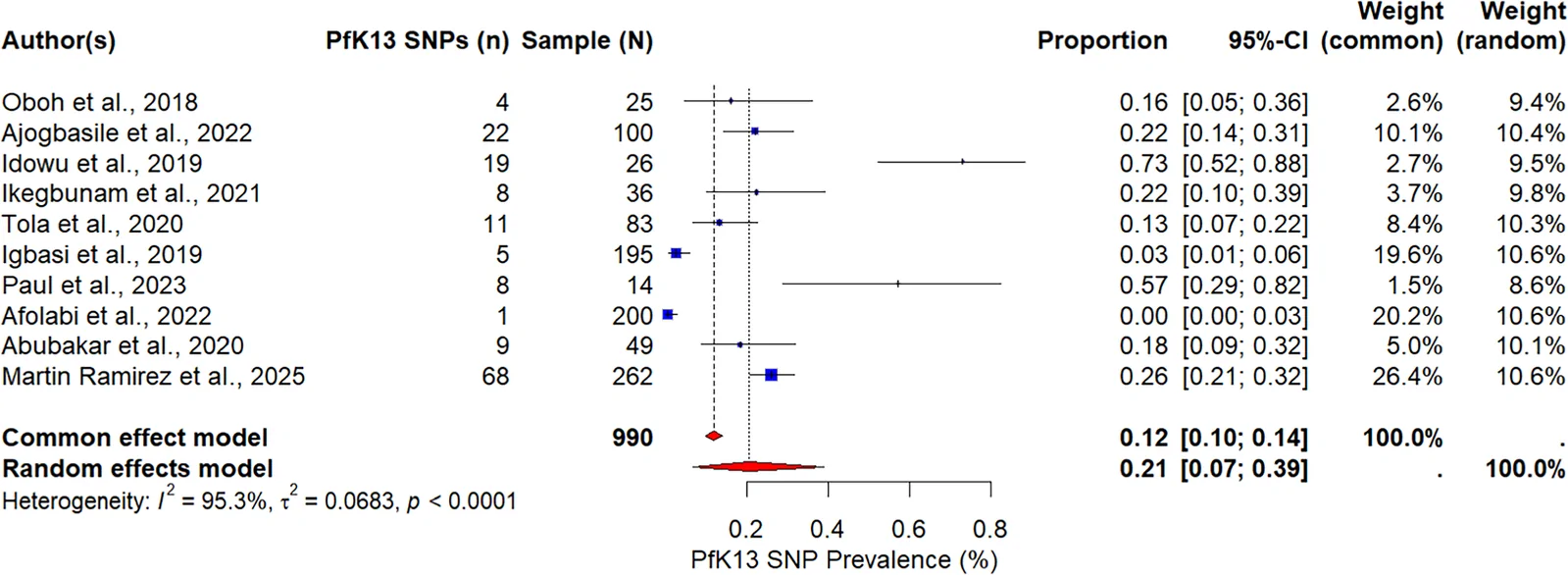
Pooled prevalence of *P. falciparum K13* SNPs from studies in Nigeria

Ten studies comprising a total of 990 samples were included in the meta-analysis of *PfK13* SNPs prevalence in Nigeria (Fig. 5). Reported prevalence estimates varied widely, ranging from 0.5% [60] to 73% [59]. The highest weighted studies under the common effect model were [56] and [60], each contributing more than 20% to the pooled estimate due to their larger sample sizes. In contrast, smaller studies [59, 61, 62] contributed less weight and displayed greater variability in their prevalence estimates. Furthermore, in the common effect model, the pooled prevalence was 12% (95% CI 10–14%), in the random effects model, a higher pooled estimate of 21% (95% CI 7–39%), reflecting the substantial heterogeneity between studies. Statistical assessment also confirmed very high heterogeneity ($I^2$ = 95.3%, $\tau ^2$ = 0.0683, p< 0.0001).

**Fig. 6:**
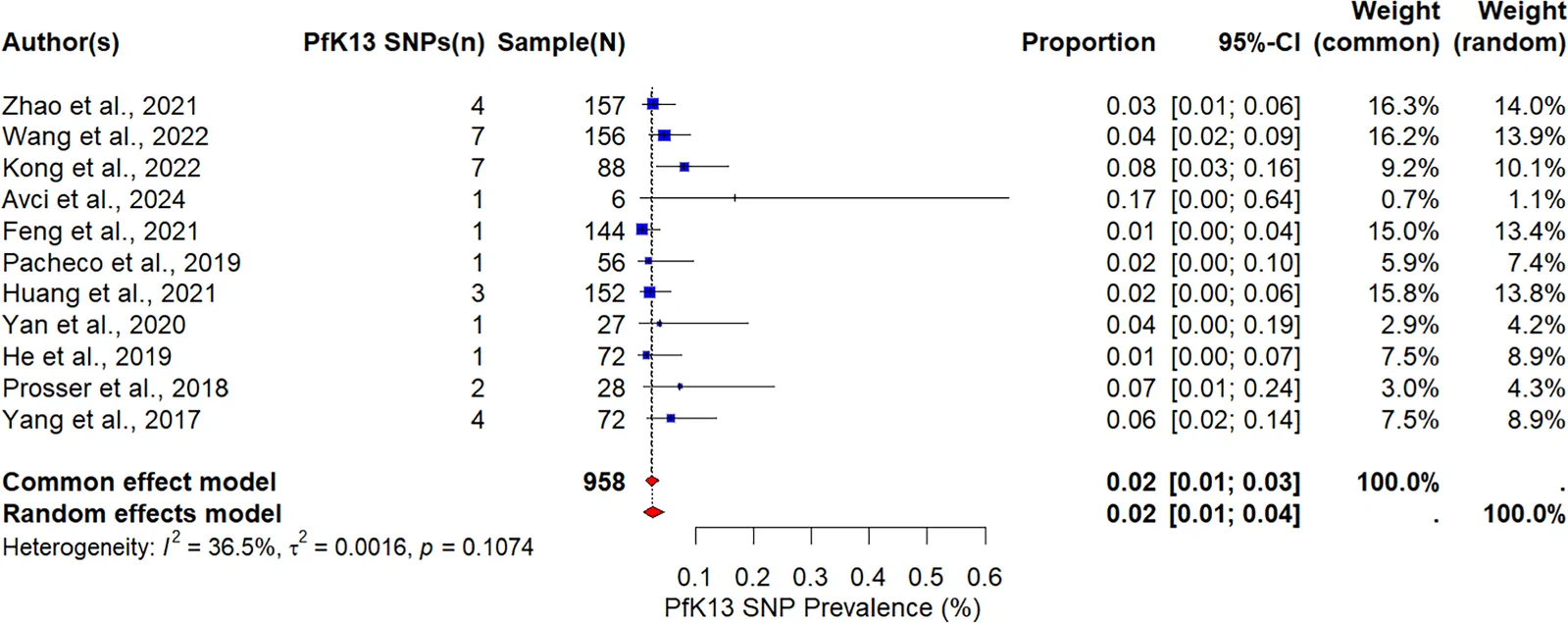
Pooled prevalence of *P. falciparum K13* SNPs of imported studies from Nigeria reported in other countries. $^{*}$ WHO-validated SNPs

In studies of imported malaria cases from Nigeria reported in other countries, a total of eleven studies, comprising 958 samples, were included in the meta-analysis to estimate the pooled prevalence of *PfK13* SNPs (Fig. 6). Individual study prevalence estimates ranged from 1% [63, 64] to 17% [65]. Larger studies such as [49, 53], and [66] demonstrated narrow CIs and contributed a greater weight to the pooled estimates, while studies with small sample sizes had wider CIs and lower weight. The common effect model showed that the pooled prevalence of the studies was 2% (95% CI 1–3%), while the random effects model produced an identical point estimate of 2% (95% CI 1–4%). The heterogeneity statistics between the studies also showed a non-significant result ($I^2$ = 36.5%, $\tau ^2$= 0.0016, *p* = 0.1074).

In the ten studies conducted in Nigeria, different *PfK13* SNPs were reported 7. The pooled prevalence under the random effect model was 0.00% (95% CI 0.00–0.00) with a heterogeneity ($I^2$ = 36.0%, $\tau ^2<$ 0.0001, p<0.0001). Individual SNPs from each study were uniformly low (≤ 7%), with only K189T deviating from the expected pooled mean value with a prevalence of 65% reported in Lagos [59]. While in the Nigerian imported malaria cases of eleven studies conducted between 2017 and 2024 in China, Turkey, the United States, and Australia (Fig. 8), the pooled prevalence under the random-effects model was 0.01% (95% CI 0.00–0.01) with no heterogeneity ($I^2$ = 0.0%, $\tau ^2<$ 0.0001, *p* = 0.9795). Individual study estimates were uniformly low (≤0.04), except for the highest point estimate (0.17) reported from a small-sample Turkish study [65].

**Fig. 7:**
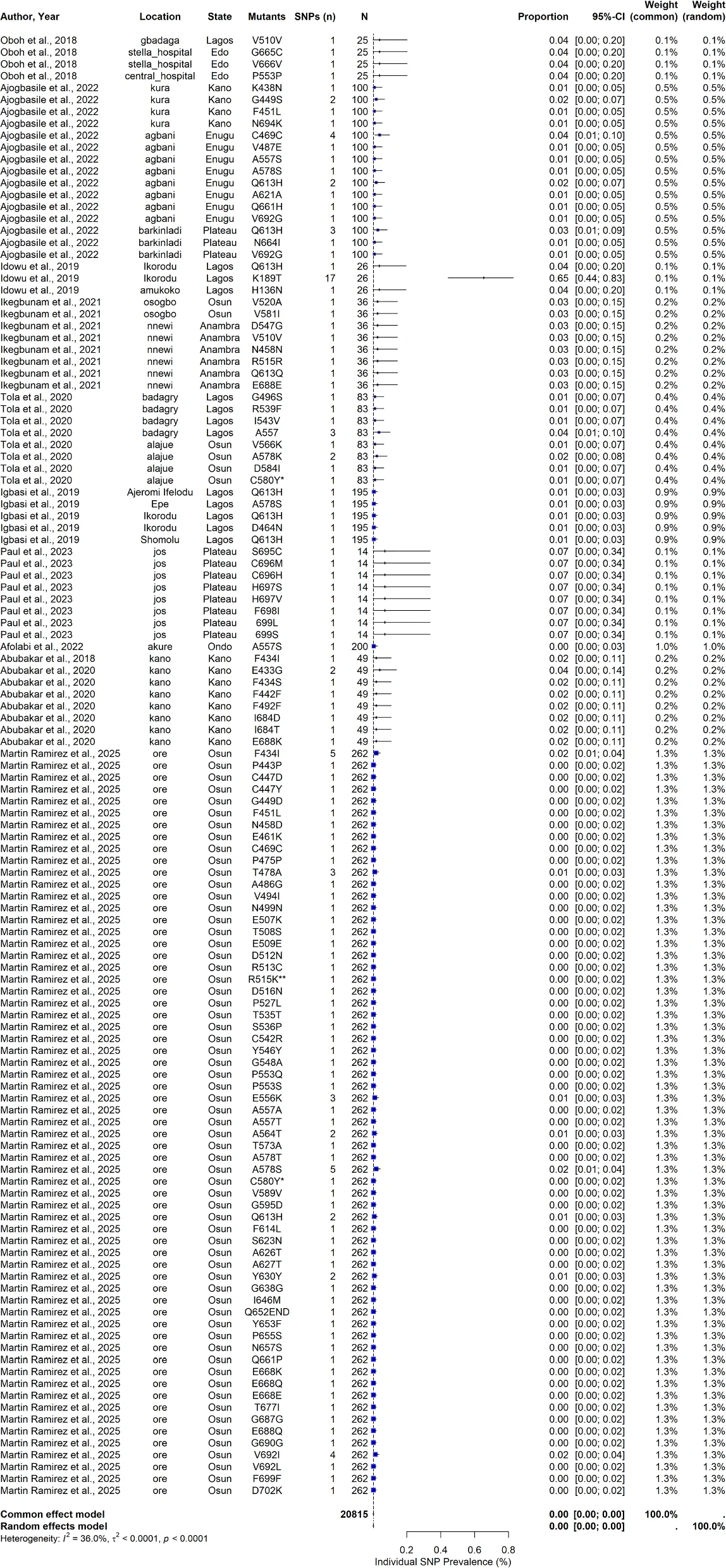
Occurrence per study of individual *P. falciparum K13* SNPs in Nigeria. Note that in computing the occurrence of the SNPs, the individual mutants from each study were repeatedly divided by the sample size, N, of their respective study. Therefore, the total sample size, N, still remains 990 (Fig. 5) and not “20815” as presented in this figure. $$^{*}$$ WHO-validated SNPs. $$^{**}$$ WHO candidate SNPs

**Fig. 8:**
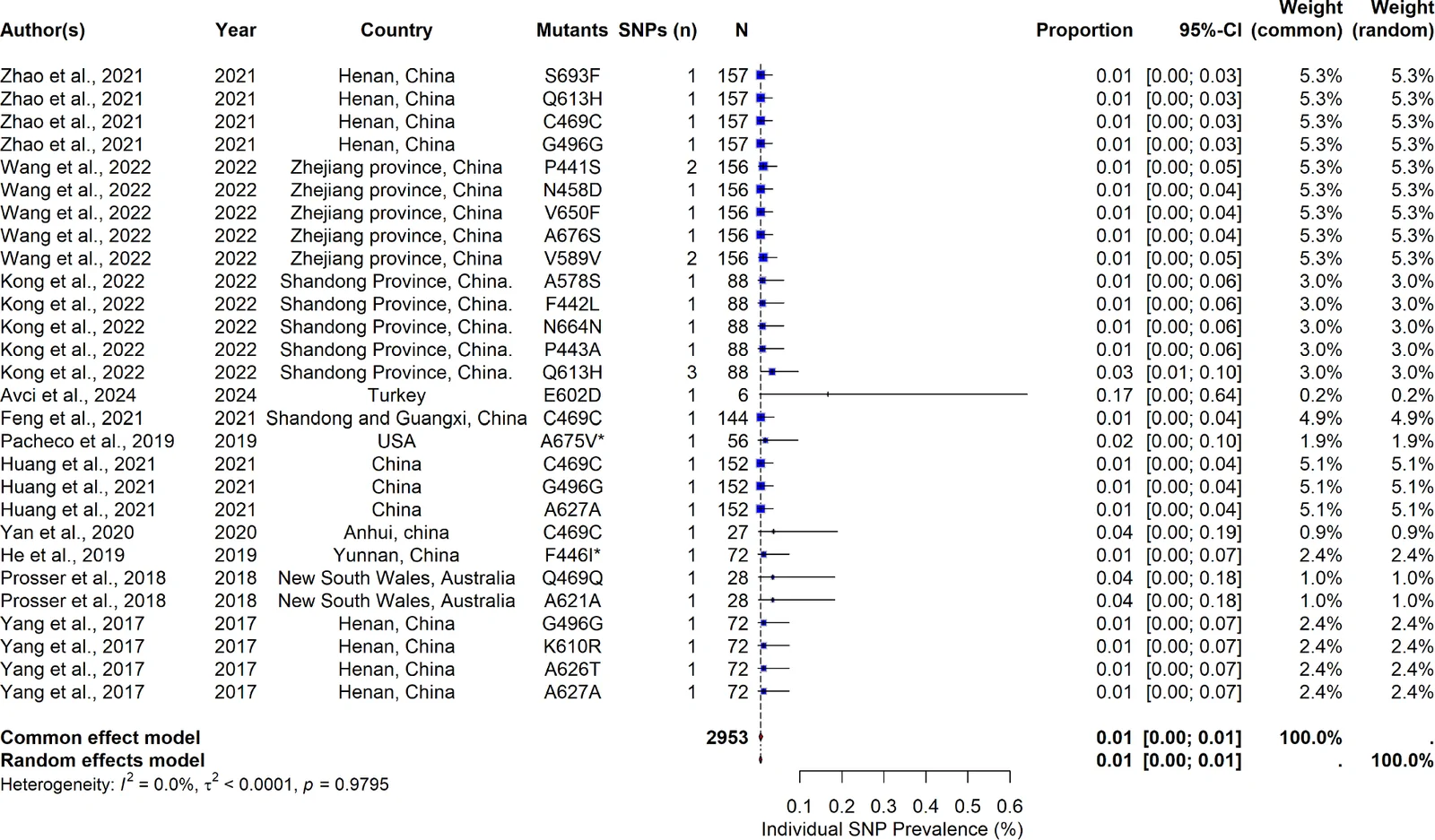
Occurrence per study of *P. falciparum K13* SNPs based on imported studies from Nigeria reported in other countries. Note that in computing the occurrence of the SNPs, the individual mutants from each study were repeatedly divided by the sample size, N, of their respective study. Therefore, the total sample size, N, still remains 958 (Fig. 6) and not “2953” as presented in this figure. $^{*}$ WHO validated SNPs

### Spatial distribution of reported *PfK13* SNPs in Nigeria

**Fig. 9:**
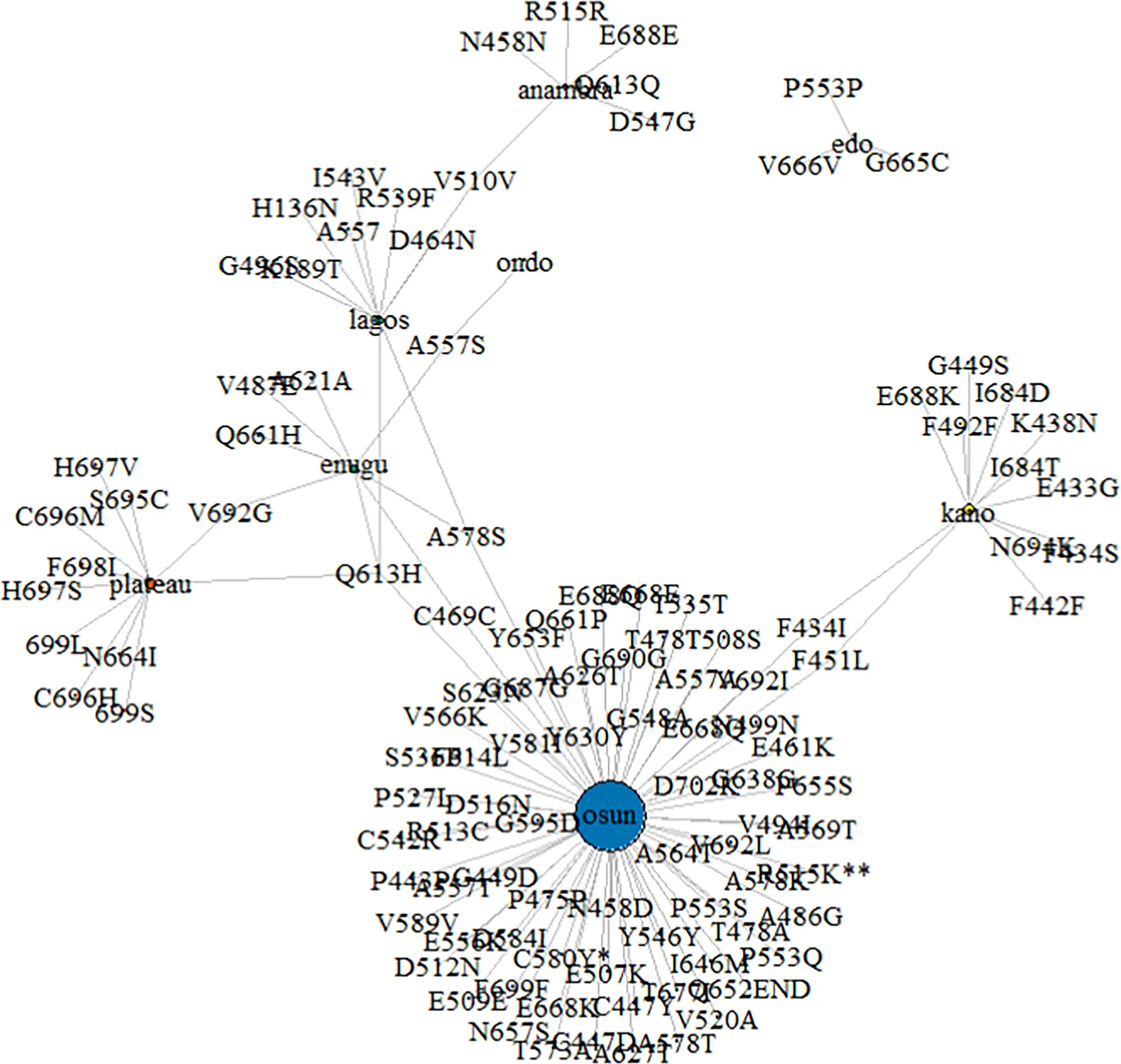
Mutation network showing the *PfK13* SNPs across different states in Nigeria where *PfK13* studies have been conducted. $$^{*}$$ WHO-validated SNPs. $$^{**}$$ WHO candidate SNPs

**Fig. 10:**
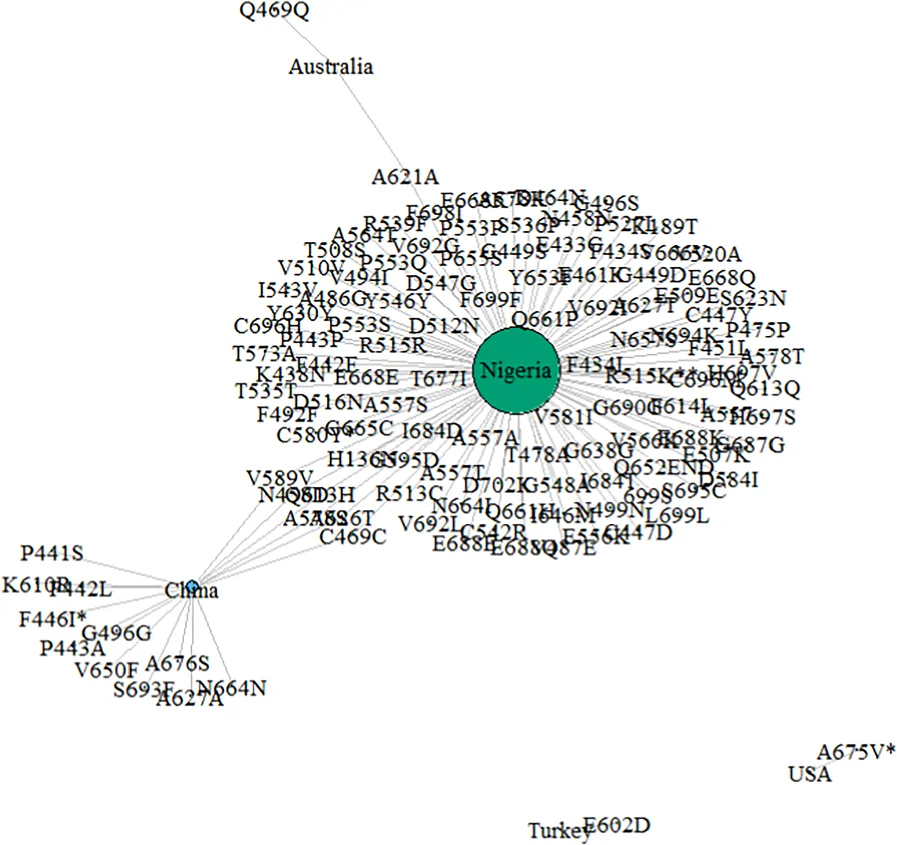
Mutation network showing the *PfK13* SNPs in studies conducted in Nigeria and studies of imported cases from Nigeria reported in other countries. $^{*}$ WHO-validated SNPs

The *P. falciparum* SNPs co-occurrence network comprised 118 nodes and 121 edges, yielding a density (0.018), indicative of sparse interconnections between mutations (Fig. 9). The diameter was 8, and the average path length was 3.35, suggesting that while most mutations are connected through relatively few intermediaries, the overall network remains loosely linked with Q613H, A578S, V510V, V692G, A557S, C469C, F434I, and F451L tied between Lagos, Enugu, Osun, and Plateau States; Lagos, Osun, and Enugu States; Lagos and Anambra States; Enugu and Plateau States; Enugu and Ondo States; Osun and Enugu States; Osun and Kano States; and finally, between Osun and Kano States, respectively. The Louvain community detection identified six distinct clusters; the modularity index was relatively high (0.571), indicating that SNPs tend to form tightly connected clusters around specific states, with limited interconnections between SNPs reported from different geographical regions like Edo, and Anambra States. Component analysis further revealed two disconnected sub-networks, indicating the presence of isolated mutation groups with no observed co-occurrence ties to the broader network, similar to what was obtained between studies in Nigeria and imported *PfK13* SNPs from Nigeria reported in other countries (Fig. 10), signifying a collectively fragmented mutational landscape.

**Table 2:** Linear regression coefficients for the association between the geographic distance (*x*_1_) and the SNPs distribution

Term	Estimate	Pr(≤b)	Pr(≥b)	Pr(≥|b|)	p-value$^\dagger$
Intercept	0.1731895265	0.685	0.315	0.475	0.475
$x_{1}$	0.0003539087	0.733	0.267	0.508	0.508

*Residual SE* = 0.4685 (df = 54); *F*-statistic = 1.639 (1, 54 df), p=0.206; $R^{2}$ = 0.02946; Adjusted $R^{2}$ = 0.01148

$^\dagger$Two-tailed *p*-value from Pr(≥|b|

**Fig. 11:**
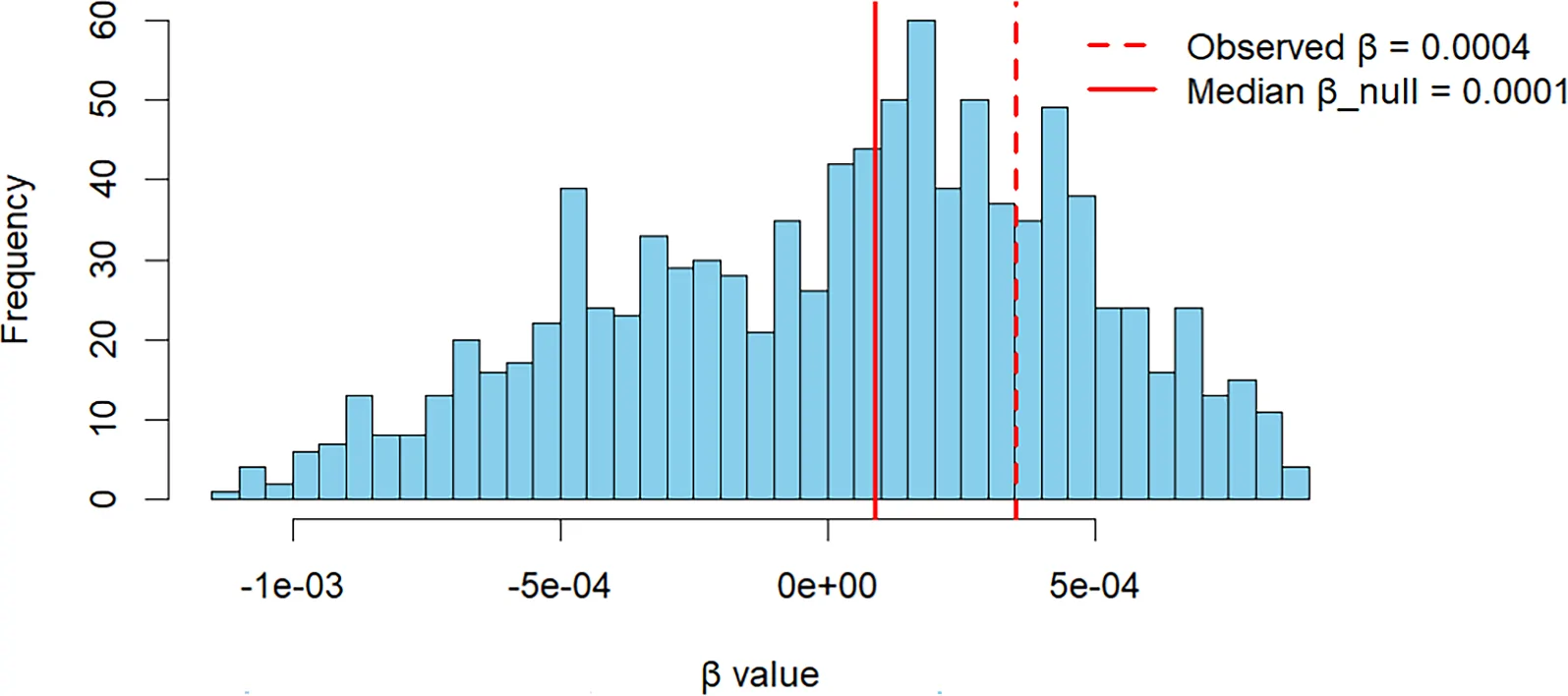
Quadratic assignment procedure (QAP) illustrating the null distribution of β values. The solid red line represents the median β under the null hypothesis, while the dashed red line indicates the observed β value

Furthermore, Table 2 showed the prediction between the geographic distance for each reporting state, taken from the respective state’s capital, $x_1$ to the co-occurrence frequency of the *PfK13* SNPs in the eight states using the OLS network model. The OLS network model yielded a small, non-significant positive coefficient for the distance (*b* = 0.000354, *p* = 0.508), indicating no detectable association between the spatial separation of states and the similarity of their SNP profiles. The intercept (*b* = 0.173, *p* = 0.475) was also non-significant. The model’s explanatory power (2.9%) was minimal (adjusted $R^2$ = 0.0115) with an overall F-statistic of 1.639 (*p* = 0.206). The quadratic assignment procedure (QAP) (Fig. 11) with 1,000 permutations further supported the absence of spatial structure, with the observed coefficient (β = 0.000354, *p*= 0.473) lying near the centre of the null distribution (median = $8.9 \times 10^{-5}$; IQR = –0.0003 to 0.0004). These findings suggest that the distribution of the observed SNP variants is unlikely to be constrained by distance, thus supporting the independent emergence of *PfK13* SNPs in the country.

### Diversity of reported *PfK13* SNPs in Nigeria

**Table 3:** *PfK13* SNPs diversity from reviewed studies

Measure of diversity	Value	p-value
Nucleotide Diversity (π)	2.307504	−
Tajima’s D	-2.593061	0.00951259
Fu & Li’s D	2.912054	0.003590606
Fu & Li’s F	0.2376935	0.8121188

Analysis of *PfK13* SNPs (table 3) revealed high nucleotide diversity (π = 2.31), indicative of substantial allelic variation in the reported *PfK13* SNPs. Tajima’s D was significantly negative (−2.59, *p* = 0.0095), consistent with an excess of rare alleles, a pattern suggestive of recent positive selection, which could be potentially driven by artemisinin drug pressure. Significant Fu & Li’s D (2.91, *p* = 0.0036) suggests that some high-frequency-derived alleles, for example, the non-synonymous Q613H and A578S, could persist in the population. However, the non-significant Fu & Li’s F (0.24, *p* = 0.81) test indicates no strong deviation from neutrality. The combined results point to a recent selective sweep in the *PfK13* gene with the persistence of some residual SNPs (e.g., Q613H and A578S) in the population.

### Geostatistical model for *PfK13* SNPs in Nigeria

**Fig. 12:**
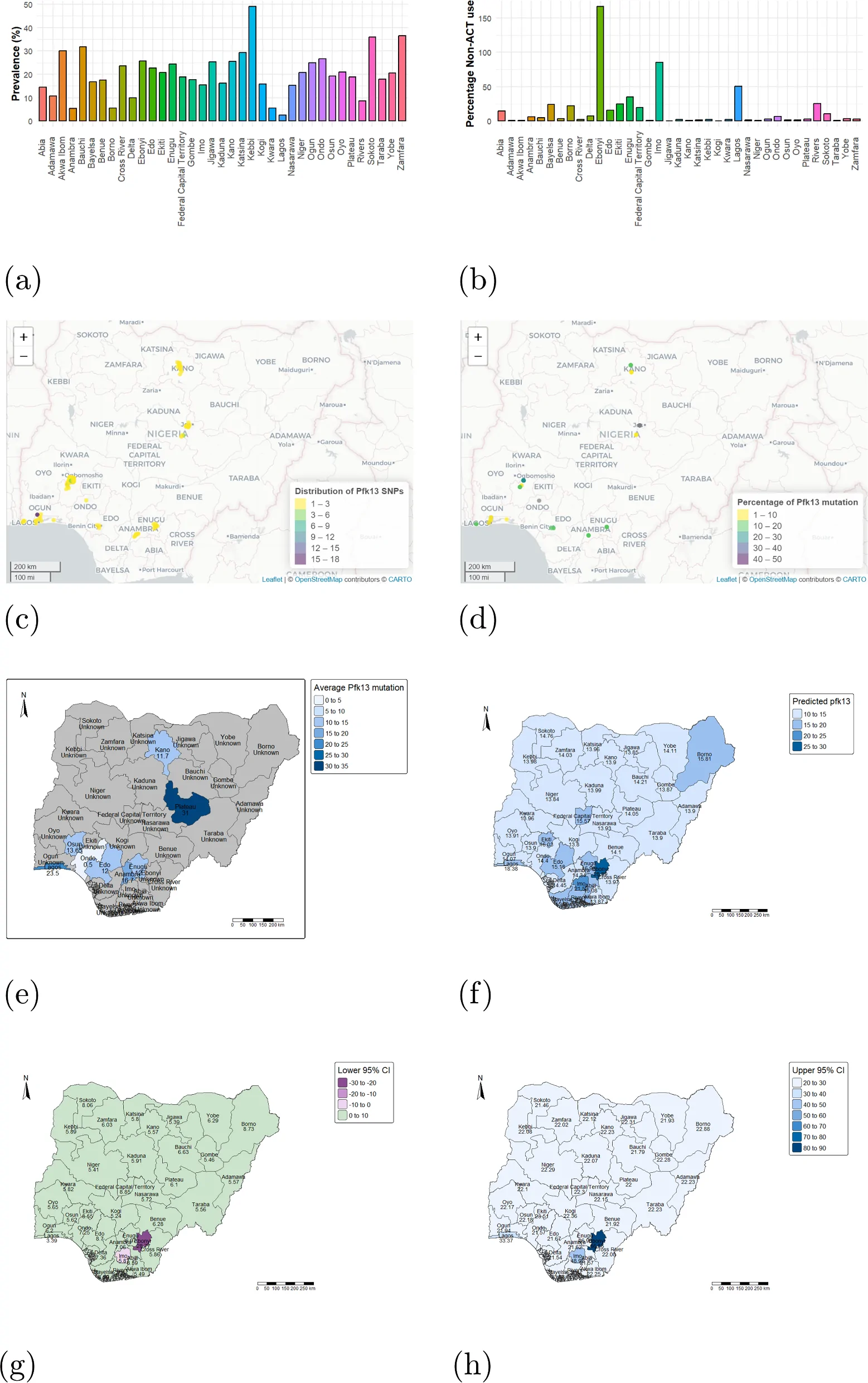
Prediction of *PfK13* mutation frequency across states in Nigeria. **a**: Malaria prevalence from the 2021 NMIS report was used as covariate. **b**: Non-ACT usage was used as an additional covariate for predicting *Pfk13* SNP prevalence. **c**: Point map of *PfK13* SNPs mutations from states where *PfK13* studies were conducted. **d**: Combined *PfK13* mutation frequency from individual studies across the states. **e**: Pooled *PfK13* SNPs prevalence from all the studies per state where *PfK13* mutation studies were carried out. **f**: Predicted *PfK13* mutation across the states in Nigeria. **g**: Lower 95% CI. **h**: Upper 95% CI. The occurrence of negative values in Imo and Eboyi States in Fig. 12g was purely as a result of the computation for the lower bound interval and is not to suggest that mutations in a population can become negative. Therefore, this is reported as “zero” in the result section

The prediction of the occurrence of mutations in the *Pfk13* gene showed to what level the gene is probably undergoing changes in Nigeria, Fig. 12. The point map, Fig. 12c, showed that most of the *PfK13* SNPs were reported from studies conducted in Lagos and Osun States. Similarly, Lagos and Osun have the most studies, followed by Kano and Plateau States. Anambra, Enugu, Edo, and Ondo States have one study each (Fig. 12d). The prevalence of the pooled *Pfk13* SNPs in all the studies for each state (Fig. 12e) was highest in Plateau (31%) and Lagos (23.5%) States and lowest in Ondo State (0.5%). Although the high prevalence recorded in Plateau State is based on a single study by [62].

The average predicted prevalence of *Pfk13* SNPs was highest in Lagos State, 18.38%, (95% CI 3.39–33.37%) and the Southeast region, e.g., Eboyi State, 28.89%, (95% CI 0–86.56%), Imo State, 21.52%, (95% CI 0–48.92%), and Enugu State 16.96%, (95% CI 6.97–26.95%), and the South-South region, e.g., Rivers State, 16.09%, (95% CI 3.48–29.7%), Bayelsa State, 15.99%, (95% CI 8.59–23.38%). Generally, the northern part of the country was predicted to have a marginally lower prevalence of the *Pfk13* mutation.

## Discussion

The Nigeria Malaria Elimination Programme is expected to kick-start the 6th National Malaria Strategic Plan by 2026, and its main objective is to eliminate malaria by 2030 [2] in accordance with the WHO global strategic agenda [67]. Progress towards achieving this plan will be highly dependent on the continued efficacy of ACTs in the country for which our review showed that resistance-validated *Pfk13* SNPs to artemisinin have been reported not just in Africa [7] but recently in Nigeria [56]. The results of the meta-analysis revealed a large divergence between the common and random effect models and also an extremely high heterogeneity, indicating that the included studies capture a highly variable epidemiological context in the occurrence of *PfK13* SNPs. The non-synonymous SNPs Q613H and A578S were the most prominent and diverse SNPs of the Nigerian study located within the propeller region. The SNPs that occur the most in the imported studies from Nigeria reported in other countries were the synonymous C469C and G496G; and the non-synonymous Q613H SNPs. The synonymous SNP C469C has been reported to be associated with late parasitological failure in Nigeria; likewise, three novel variants (V692G, N664I, and Q661H) in Enugu and Plateau States [68]; the C469C was also reported in imported SNPs from Nigeria [64]. The non-synonymous A578S SNP is the most reported SNP in Africa [17], this was also reported in the study conducted in Nigeria by [68] in Enugu State, by [69] in Lagos State, and by [56] in Osun State and also in the imported study reported by [70] in Shandong, China. However, this SNP has not been associated with artemisinin resistance, as evidenced by ring-stage in vitro survival assays [17].

Studies by [56, 59, 62, 68, 71] reported the prevalence of *PfK13* SNPs exceeding 20%. This may be driven by localized transmission dynamics, as many SNPs identified in the country are novel SNPs [68]. In contrast, studies with larger sample sizes present lower prevalence [60, 69] and had narrower confidence intervals, dragging the common effect estimate downward. These findings highlight the risk of underestimating the prevalence of *PfK13* SNPs and underscore the need for geographically targeted surveillance. Such targeted approaches could help identify and contain emerging resistance SNPs before they spread to broader areas.

The pooled *PfK13* SNPs prevalence of approximately 2% observed in the meta-analysis of studies from Nigeria reported in other countries suggests that *PfK13* mutations remain relatively rare in the study population. The consistency between the common- and random-effects models, combined with the low heterogeneity, reinforces the robustness of the estimate. This is as expected because these countries do not have active malaria transmission locally [72], unlike in endemic countries, where the use of ACTs or artemisinin monotherapy due to malaria infection could result in the emergence of artemisinin-resistant alleles of *P. falciparum* malaria parasites [14]. A major limitation to the heterogeneity and spatial distribution model estimates is the overall rare occurrence of SNPs *PfK13*, both in the domestic studies and in imported malaria cases from Nigeria reported in other countries in this review. However, the presence of SNPs, even at low frequency, warrants continued monitoring, as localized increases could signal the early emergence of resistant parasite strains.

The temporal trends showed an increasing frequency of reported *PfK13* polymorphisms over the review period, particularly among studies conducted within Nigeria. The odds of detecting a reported *PfK13* polymorphism increased by approximately 18% per year in Nigerian studies, although the observed annual frequencies were heterogeneous. Imported cases from Nigeria identified outside the country may differ from infections sampled within the country. This difference may reflect the selective nature of imported cases which may originate from specific geographic regions or populations rather than the country as a whole. Differences in sample size, clinical presentation, parasite selection, and molecular methods may also contribute to the observed variation [73]. The absence of a significant temporal trend among imported cases therefore does not necessarily contradict the increasing trend observed in Nigerian studies. Also, the significant overall temporal association should not be interpreted as evidence that the introduction of ACTs in 2005 [26] caused an increase in *PfK13* polymorphisms. The reason being that available molecular studies in this review began in 2017, leaving no comparable pre-ACT *PfK13* observations against which to assess a true pre- and post-ACT change. Nevertheless, the increasing trend among Nigerian studies highlights the importance of sustained molecular surveillance markers associated with artemisinin susceptibility.

In Nigeria, studies on *P. falciparum* kelch13 SNPs were only obtained for eight states of the 36 states and the Federal Capital Territory (FCT), with considerable variation in the mutations identified across locations. The mutation network showed little overlap between states, while Edo State had no connection with any other state, suggesting that some mutations may be emerging independently. This diversity highlights the dynamic and regionally variable nature of kelch13 variation in Nigeria and may reflect differences in transmission patterns, antimalarial drug use, treatment practices, and parasite population structure [74]. The absence of a single dominant mutation across the reported studies could also suggest localized evolutionary or selection pressures, although differences in sampling and surveillance coverage may partly explain the observed pattern [75]. Overall, these findings underscore the need for broader and more geographically representative molecular surveillance of kelch13 variants across Nigeria.

These findings indicate an exceptionally low circulation of *PfK13* mutations across the surveyed populations, with no evidence of regional clustering in states or emerging hot spots. Most detected variants, such as K189T, Q613H, A578S, G496G, and C469C, are not validated markers of resistance to artemisinin, suggesting limited clinical relevance. This supports ACT’s continued effectiveness in treating uncomplicated malaria cases in Nigeria. However, the geographic breadth of detection underscores the need for sustained genomic surveillance to detect early expansion of resistance-associated alleles before they reach significant epidemiological levels. The recent confirmatory report of WHO-validated C580Y [56], which was previously suspected by [76], and the candidate SNP R515K [56], reinforces the need for sustained genomic surveillance in Nigeria. Again, WHO-validated SNPs F446I [63] and A675V [58] were reported in Yunnan, China, and the USA, respectively, in imported cases from Nigeria. A wide range of *Pfk13* WHO validated and candidate SNPs has been reported from various African studies, particularly in east Africa. For example, R561H, A675V, and C469Y was reported in Uganda; A675V, C469Y, V568G, P574L, P553L, and R561H in Kenya; R622I and C580Y in Ethiopia; R561H, A675V, P574L, C469F, and P441L in Rwanda; R561H, R622I, and A675V in Tanzania; and R622I in Eritrea[6]. in west Africa, P553L has been reported in Angola [43]; and P574L, R561H, N537I, C469F, C469Y, P553L, P441L, and A675V in Ghana [6]. Among the reported SNPs in Nigeria, A675V SNP imported from Nigeria into other countries was detected to be widely distributed in East Africa and has also been reported in Ghana. This SNP was reported in Ugangda, Kenya, Rwanda, and Tanzania. Also, C580Y reported by [56] from isolates in Nigeria has also been reported in Ethiopia [6].

The independent emergence of mutations in the *PfK13* gene has been widely reported, especially in African studies [17, 59, 77, 78]. Unlike what happened with the other antimalarial resistance markers, where mutations spread from the SEA to Africa [15], SNPs of *PfK13* have been reported to emerge locally [14, 68]. The network analysis showed the lack of linkages between SNPs from different states, with the spatial distance between states having no effect on the similarity of the SNPs reported in this review. Furthermore, the diversity of SNPs was high with few *PfK13* mutations per study, consistent with studies conducted in Africa [17]. Unlike the situation in SEA, with a low *PfK13* SNPs diversity but high *Pfk13* mutation frequencies in each study, many of which are associated with resistance to artemisinin [17]. African studies have continually reported high *Pfk13* SNPs diversity, the majority of which are not validated or associated with resistance to artemisinin [17]. A recent study in northern Uganda documented a moderate prevalence of C469Y ($\approx 30.5 \%$) and A675V ($\approx 8.5 \%$) both validated markers beginning in 2021 and remaining stable over time, with evidence that these polymorphisms are associated with reduced *in vitro* susceptibility to dihydroartemisinin and lumefantrine [79]. In Rwanda, the R561H mutation, another validated marker, has emerged at high frequency and confers elevated survival *in vitro*, comparable to C580Y; this mutation appears epidemiologically independent of the Southeast Asian lineages [23]. Furthermore, a continent-wide meta-analysis spanning 29 African countries identified validated *PfK13* markers, including R561H in Rwanda and Tanzania, F446I in Mali, C580Y in Ghana, P553L in Angola, and M476I in Tanzania with a low but widespread distribution [7]; however, despite these mutations, ACTs continue to remain effective in Africa [3].

The low reports of WHO-validated SNPs in endemic countries such as Nigeria could be due to the few studies that have been conducted in the country with small sample sizes and perhaps due to the sequencing approach used [59]. Until recently [56], reported the presence of C580Y and R515K, no validated or candidate SNPs have been reported in Nigeria. Furthermore, the results of two studies [63] and [58] of imported malaria from Nigeria reported in other countries identified the presence of validated SNPs, F446I and A675V respectively. This further established the fact that *PfK13* SNPs known to cause artemisinin resistance are already in circulation in the country, although this could be at a low frequency.

The conventional approach to identifying resistant parasite strains is based on *in vivo* and *in vitro* drug susceptibility assays [17]. Although these methods remain effective, they are constrained by several limitations, most notably their high cost and labor-intensive nature [8]. In contrast, molecular resistance markers provide a more efficient and scalable alternative, allowing not only the detection of resistant strains but also the prediction of their emergence and spread [15]. In this review, the occurrence of the *PfK13* SNP in states where *PfK13* studies have not been conducted was predicted. The prediction revealed higher mutation frequencies in Lagos and the Southeast and South-South regions of the country. This prediction could be used to make informed decisions on which state samples could be collected for *Pfk13* genotyping and therapeutic efficacy studies (TES), and it may also serve as a useful guide for policy decisions on the use of ACTs in different parts of the country. Parasite prevalence and the use of other antimalarial drugs (monotherapies) were included as predictors based on previous evidence suggesting that: (i) host immunity, which is higher in highly endemic regions, can facilitate clearance of partially resistant parasites, thereby reducing the likelihood of resistant strains becoming fixed [30, 80]; (ii) in high-transmission areas, the concurrent transmission of multiple *P. falciparum* strains to mosquito vectors increases opportunities for genetic recombination during meiosis, which can disrupt resistance alleles and promote parasite diversity, leading to competition that limits fixation of resistant variants [14, 80]; and (iii) in low-transmission settings, where multiple infections are less common and host immunity is lower, individuals are more likely to experience symptomatic infections and therefore more frequently undergo treatment, which might be with poor-quality antimalarials, incomplete treatment courses, or monotherapies which may include artemisinin, all of which can contribute to the emergence and fixation of resistant parasites [14]. A major limitation to this prediction is that the covariates used may not fully capture the complexity of the factors that drive the emergence of *PfK13* mutations. Therefore, the prediction should be interpreted with caution.

Despite this limitation, this study provides useful insights on the potential distribution of *PfK13* mutations across Nigeria. In addition, predictive approaches of this kind could serve as valuable tools for epidemiological studies in tracking the spread of drug-resistant mutations. These models could be further strengthened by incorporating additional covariates, such as the prevalence of falsified and substandard ACTs that contain less active ingredients, which remain a critical driver of resistance [14].

Generally, ACTs continue to demonstrate high efficacy in Africa, including Nigeria, where several TES have consistently reported cure rates exceeding 95% [27, 81–83]. These findings reinforce the continued role of ACTs as the cornerstone of malaria case management in the country. However, emerging evidence of validated and candidate *Pfk13* SNPs is beginning to raise concern [56]. Reports of validated and candidate *PfK13* SNPs associated with partial artemisinin resistance have been documented in multiple African countries, highlighting the potential for independent emergence or importation of resistant parasite lineages [80]. Of particular concern is the detection of C580Y and R515K polymorphisms in studies conducted in Nigeria [56]; and F446I and A675V from imported SNPs in Nigeria reported in other countries [58, 63], as presented in this review study, which mirror mutations previously associated with delayed parasite clearance in Southeast Asia [17]. Although their clinical significance in Africa remains under investigation, their presence signals that alleles known to cause resistance are already in circulation in the Nigerian *P. falciparum* population.

These findings highlight the need to integrate molecular *PfK13* surveillance with therapeutic efficacy monitoring to provide early warning of emerging artemisinin resistance before it becomes widespread and inform future antimalarial treatment policies in Nigeria, while avoiding premature changes to ACT policy based solely on molecular polymorphism data. Such surveillance would provide actionable information for national malaria control programs, enabling evidence-based decisions on treatment policies and supporting the timely deployment of resistance mitigation strategies. These may include the adoption of multiple first-line ACT regimens [84], the evaluation of triple ACT combinations designed to delay resistance selection [85], and the accelerated development and clinical testing of novel antimalarial combinations [3]. Proactive integration of molecular epidemiology into malaria control frameworks will be critical to safeguarding the efficacy of ACTs and sustaining the progress made toward malaria elimination in Nigeria and in sub-Saharan Africa.

## Conclusion

The study reveals a remarkable genetic diversity in the Kelch13 gene of *P. falciparum* in Nigeria. The pronounced diversity in the kelch13 SNP underscores the dynamic and regionally variable nature of the *P. falciparum* kelch13 SNP within the country. Despite this diversity, no single mutation appears to dominate in all studies, suggesting the absence of uniform selection pressure or widespread fixation of polymorphic *Pfk13* alleles. Spatial network modelling further indicates that the distribution of *Pfk13* variants is not geographically structured, suggesting that *PfK13* mutations could be emerging independently in different states. The report of validated and candidate *Pfk13* SNPs highlights an evolving molecular landscape that warrants close surveillance. The marginally higher predicted prevalence of *Pfk13* mutations in the Southeast, South-South, and Lagos State may signal emerging regional hotspots. Collectively, these findings emphasize the necessity for continuous molecular surveillance and integrative epidemiogenomic monitoring to anticipate and mitigate the potential spread of artemisinin and partner drug resistance in the country.

## Supplementary Information

Supplementary Material 1

## Data Availability

The Ungdata surveys that support the findings of this study are available from Norwegian Social Research (NOVA), but restrictions apply to the availability of these data, which were used under licence for the current study and so are not publicly available. The data are, however, available upon request and with the permission of Norwegian Social Research (NOVA). All data supporting the findings of this study are available within the paper and its Supplementary Information. Microsatellite primer sequences are provided in Supplementary Table 2, along with original reference describing the microsatellites used in this study.
