# Supplementary material for: Evolving genetic landscapes of Plasmodium falciparum Kelch13 polymorphisms in Nigeria: a systematic review and meta-analysis
Source: Malar J. 2026 Sep 21;25:381. doi: 10.1186/s12936-026-06138-x (PMC13711917; doi:10.1186/s12936-026-06138-x)
Supplement: Supplementary file 1 [file 12936_2026_6138_MOESM1_ESM.pdf]

Evolving genetic landscapes of *Plasmodium falciparum* Kelch13 polymorphisms in Nigeria: A systematic review and meta-analysis (Supplementary)

nwanaaaron152

July 2025

Table 1: Search strategy and databases used in the systematic review

Database	Search strategy
**PubMed**	1) Naive search: Artemisinin, *PfK13*, Nigeria<br>2) Secondary search term generated: (artemisinin OR "drug resistance" OR resistance) AND (*Pfk13* OR genes) AND (nigeria) AND (malaria OR falciparum OR plasmodium)<br>3) Custom range: 2005-2024
**Springer Nature**	1) Naive search: Artemisinin, *PfK13*, Nigeria<br>2) Content type: Articles; Custom dates: 2005-2024; Language: English; Subject: Malaria
**Google Scholar**	1) Naive search (in the advanced search section): Artemisinin, *PfK13*, Nigeria<br>2) Find articles: With all the words (Artemisinin AND *PfK13* AND Nigeria)<br>3) Where my words occur: Anywhere in the article<br>4) Return articles dated between 2005 and 2024.

Table 2: Summary of studies reporting *PfK13* polymorphisms in Nigeria

s/n	ID	Author	Location	State	N	SNPs	Sample size	Domain (441 to 726)	Syn /Non-syn	Lat	Lon
1	A	[1]	gbadaga	lagos	1	V510V	25	1	1	6.5569568	3.380168
2	B	[1]	stella_hospital	edo	1	G665C	25	1	0	6.2747161	5.6289076
3	C	[1]	stella_hospital	edo	1	V666V	25	1	1	6.2747161	5.6289076
4	D	[1]	central_hospital	edo	1	P553P	25	1	1	6.2748857	5.549079
5	E	[2]	kura	kano	1	K438N	100	0	0	11.7721673	8.4167477
6	F	[2]	kura	kano	1	G449S	100	1	0	11.7721673	8.4167477
7	G	[2]	kura	kano	1	G449S	100	1	0	11.7721673	8.4167477
8	H	[2]	kura	kano	1	F451L	100	1	0	11.7721673	8.4167477
9	I	[2]	kura	kano	1	N694K	100	1	0	11.7721673	8.4167477
10	J	[2]	agbani	enugu	1	C469C	100	1	1	6.3106608	7.5316967
11	K	[2]	agbani	enugu	1	C469C	100	1	1	6.3106608	7.5316967
12	L	[2]	agbani	enugu	1	C469C	100	1	1	6.3106608	7.5316967
13	M	[2]	agbani	enugu	1	C469C	100	1	1	6.3106608	7.5316967
14	N	[2]	agbani	enugu	1	V487E	100	1	0	6.3106608	7.5316967
15	O	[2]	agbani	enugu	1	A557S	100	1	0	6.3106608	7.5316967
16	P	[2]	agbani	enugu	1	A578S	100	1	0	6.3106608	7.5316967
17	Q	[2]	agbani	enugu	1	Q613H	100	1	0	6.3106608	7.5316967
18	R	[2]	agbani	enugu	1	Q613H	100	1	0	6.3106608	7.5316967
19	S	[2]	agbani	enugu	1	A621A	100	1	1	6.3106608	7.5316967
20	T	[2]	agbani	enugu	1	Q661H	100	1	0	6.3106608	7.5316967
21	U	[2]	agbani	enugu	1	V692G	100	1	0	6.3106608	7.5316967
22	V	[2]	barkinladi	plateau	1	Q613H	100	1	0	9.5762198	8.5837032
23	W	[2]	barkinladi	plateau	1	Q613H	100	1	0	9.5762198	8.5837032
24	X	[2]	barkinladi	plateau	1	Q613H	100	1	0	9.5762198	8.5837032
25	Y	[2]	barkinladi	plateau	1	N664I	100	1	0	9.5762198	8.5837032
26	Z	[2]	barkinladi	plateau	1	V692G	100	1	0	9.5762198	8.5837032
27	AA	[3]	Ikorodu	lagos	1	Q613H	26	1	0	6.6494443	3.3943739
28	AB	[3]	Ikorodu	lagos	1	K189T	26	0	0	6.6494443	3.3943739
29	AC	[3]	Ikorodu	lagos	1	K189T	26	0	0	6.6494443	3.3943739
30	AD	[3]	Ikorodu	lagos	1	K189T	26	0	0	6.6494443	3.3943739
31	AE	[3]	Ikorodu	lagos	1	K189T	26	0	0	6.6494443	3.3943739
32	AF	[3]	Ikorodu	lagos	1	K189T	26	0	0	6.6494443	3.3943739
33	AG	[3]	Ikorodu	lagos	1	K189T	26	0	0	6.6494443	3.3943739
34	AH	[3]	Ikorodu	lagos	1	K189T	26	0	0	6.6494443	3.3943739
35	AI	[3]	Ikorodu	lagos	1	K189T	26	0	0	6.6494443	3.3943739
36	AJ	[3]	Ikorodu	lagos	1	K189T	26	0	0	6.6494443	3.3943739
37	AK	[3]	Ikorodu	lagos	1	K189T	26	0	0	6.6494443	3.3943739
38	AL	[3]	Ikorodu	lagos	1	K189T	26	0	0	6.6494443	3.3943739
39	AM	[3]	Ikorodu	lagos	1	K189T	26	0	0	6.6494443	3.3943739
40	AN	[3]	Ikorodu	lagos	1	K189T	26	0	0	6.6494443	3.3943739
41	AO	[3]	Ikorodu	lagos	1	K189T	26	0	0	6.6494443	3.3943739
42	AP	[3]	Ikorodu	lagos	1	K189T	26	0	0	6.6494443	3.3943739
43	AQ	[3]	Ikorodu	lagos	1	K189T	26	0	0	6.6494443	3.3943739
44	AR	[3]	Ikorodu	lagos	1	K189T	26	0	0	6.6494443	3.3943739
45	AS	[3]	amukoko	lagos	1	H136N	26	0	0	6.4720083	3.328466

s/n	ID	Author	Location	State	N	SNPs	Sample size	Domain (441 to 726)	Syn /Non-syn	Lat	Lon
46	AT	[4]	osogbo	osun	1	V520A	71	1	0	7.7842931	4.4802991
47	AU	[4]	osogbo	osun	1	V581I	71	1	0	7.7842931	4.4802991
48	AV	[4]	nnewi	anambra	1	D547G	36	1	0	6.0127897	6.8825267
49	AW	[4]	nnewi	anambra	1	V510V	36	1	1	6.0127897	6.8825267
50	AX	[4]	nnewi	anambra	1	N458N	36	1	1	6.0127897	6.8825267
51	AY	[4]	nnewi	anambra	1	R515R	36	1	1	6.0127897	6.8825267
52	AZ	[4]	nnewi	anambra	1	Q613Q	36	1	1	6.0127897	6.8825267
53	BA	[4]	nnewi	anambra	1	E688E	36	1	1	6.0127897	6.8825267
54	BB	[5]	badagry	lagos	1	G496S	83	1	0	6.4172387	2.8703832
55	BC	[5]	badagry	lagos	1	R539F	83	1	0	6.4172387	2.8703832
56	BD	[5]	badagry	lagos	1	I543V	83	1	0	6.4172387	2.8703832
57	BE	[5]	badagry	lagos	1	A557	83	1	0	7.7287143	4.4290555
58	BF	[5]	badagry	lagos	1	A557	83	1	0	7.7287143	4.4290555
59	BG	[5]	badagry	lagos	1	A557	83	1	0	7.7287143	4.4290555
60	BH	[5]	alajue	osun	1	V566K	83	1	0	7.7287143	4.4290555
61	BI	[5]	alajue	osun	1	A578K	83	1	0	7.7287143	4.4290555
62	BJ	[5]	alajue	osun	1	A578K	83	1	0	7.7287143	4.4290555
63	BK	[5]	alajue	osun	1	D584I	83	1	0	7.7287143	4.4290555
64	BL	[5]	alajue	osun	1	C580Y	83	1	0	7.7287143	4.4290555
65	BM	[6]	Ajeromi Ifelodu	lagos	1	Q613H	195	1	0	6.4553041	3.3153279
66	BN	[6]	Epe	lagos	1	A578S	195	1	0	6.5861787	3.9536263
67	BO	[6]	Ikorodu	lagos	1	Q613H	195	1	0	6.6494443	3.3943739
68	BP	[6]	Ikorodu	lagos	1	D464N	195	1	0	6.6494443	3.3943739
69	BQ	[6]	Shomolu	lagos	1	Q613H	195	1	0	6.5376382	3.3651074
70	BR	[7]	jos	plateau	1	S695C	14	1	0	9.9084166	8.7126166
71	BS	[7]	jos	plateau	1	C696M	14	1	0	9.9084166	8.7126166
72	BT	[7]	jos	plateau	1	C696H	14	1	0	9.9084166	8.7126166
73	BU	[7]	jos	plateau	1	H697S	14	1	0	9.9084166	8.7126166
74	BV	[7]	jos	plateau	1	H697V	14	1	0	9.9084166	8.7126166
75	BW	[7]	jos	plateau	1	F698I	14	1	0	9.9084166	8.7126166
76	BX	[7]	jos	plateau	1	699L	14	1	0	9.9084166	8.7126166
77	BY	[7]	jos	plateau	1	699S	14	1	0	9.9084166	8.7126166
78	BZ	[8]	akure	ondo	1	A557S	200	1	0	7.2461995	5.1131
79	CA	[9]	kano	kano	1	F434I	49	0	0	12.0171339	8.3919109
80	CB	[9]	kano	kano	1	E433G	49	0	0	12.0171339	8.3919109
81	CC	[9]	kano	kano	1	E433G	49	0	0	12.0171339	8.3919109
82	CD	[9]	kano	kano	1	F434S	49	0	0	12.0171339	8.3919109
83	CE	[9]	kano	kano	1	F442F	49	1	1	12.0171339	8.3919109
84	CF	[9]	kano	kano	1	F492F	49	1	1	12.0171339	8.3919109
85	CG	[9]	kano	kano	1	I684D	49	1	0	12.0171339	8.3919109
86	CH	[9]	kano	kano	1	I684T	49	1	0	12.0171339	8.3919109
87	CI	[9]	kano	kano	1	E688K	49	1	0	12.0171339	8.3919109
88	CJ	[10]	ore	osun	1	F434I	262	0	0	7.9624732	4.5576577
89	CK	[10]	ore	osun	1	F434I	262	0	0	7.9624732	4.5576577
90	CL	[10]	ore	osun	1	F434I	262	0	0	7.9624732	4.5576577
91	CM	[10]	ore	osun	1	F434I	262	0	0	7.9624732	4.5576577
92	CN	[10]	ore	osun	1	F434I	262	0	0	7.9624732	4.5576577
93	CO	[10]	ore	osun	1	P443P	262	1	1	7.9624732	4.5576577

s/n	ID	Author	Location	State	N	SNPs	Sample size	Domain (441 to 726)	Syn /Non-syn	Lat	Lon
94	CP	[10]	ore	osun	1	C447D	262	1	0	7.9624732	4.5576577
95	CQ	[10]	ore	osun	1	C447Y	262	1	0	7.9624732	4.5576577
96	CR	[10]	ore	osun	1	G449D	262	1	0	7.9624732	4.5576577
97	CS	[10]	ore	osun	1	F451L	262	1	0	7.9624732	4.5576577
98	CT	[10]	ore	osun	1	N458D	262	1	0	7.9624732	4.5576577
99	CU	[10]	ore	osun	1	E461K	262	1	0	7.9624732	4.5576577
100	CV	[10]	ore	osun	1	C469C	262	1	1	7.9624732	4.5576577
101	CW	[10]	ore	osun	1	P475P	262	1	1	7.9624732	4.5576577
102	CX	[10]	ore	osun	1	T478A	262	1	0	7.9624732	4.5576577
103	CY	[10]	ore	osun	1	T478A	262	1	0	7.9624732	4.5576577
104	CZ	[10]	ore	osun	1	T478T	262	1	1	7.9624732	4.5576577
105	DA	[10]	ore	osun	1	A486G	262	1	0	7.9624732	4.5576577
106	DB	[10]	ore	osun	1	V494I	262	1	0	7.9624732	4.5576577
107	DC	[10]	ore	osun	1	N499N	262	1	1	7.9624732	4.5576577
108	DD	[10]	ore	osun	1	E507K	262	1	0	7.9624732	4.5576577
109	DE	[10]	ore	osun	1	T508S	262	1	0	7.9624732	4.5576577
110	DF	[10]	ore	osun	1	E509E	262	1	1	7.9624732	4.5576577
111	DG	[10]	ore	osun	1	D512N	262	1	0	7.9624732	4.5576577
112	DH	[10]	ore	osun	1	R513C	262	1	0	7.9624732	4.5576577
113	DI	[10]	ore	osun	1	R515K	262	1	0	7.9624732	4.5576577
114	DJ	[10]	ore	osun	1	D516N	262	1	0	7.9624732	4.5576577
115	DK	[10]	ore	osun	1	P527L	262	1	0	7.9624732	4.5576577
116	DL	[10]	ore	osun	1	T535T	262	1	1	7.9624732	4.5576577
117	DM	[10]	ore	osun	1	S536P	262	1	0	7.9624732	4.5576577
118	DN	[10]	ore	osun	1	C542R	262	1	0	7.9624732	4.5576577
119	DO	[10]	ore	osun	1	Y546Y	262	1	1	7.9624732	4.5576577
120	DP	[10]	ore	osun	1	G548A	262	1	0	7.9624732	4.5576577
121	DQ	[10]	ore	osun	1	P553Q	262	1	0	7.9624732	4.5576577
122	DR	[10]	ore	osun	1	P553S	262	1	0	7.9624732	4.5576577
123	DS	[10]	ore	osun	1	E556K	262	1	0	7.9624732	4.5576577
124	DT	[10]	ore	osun	1	E556K	262	1	0	7.9624732	4.5576577
125	DU	[10]	ore	osun	1	E556K	262	1	0	7.9624732	4.5576577
126	DV	[10]	ore	osun	1	A557A	262	1	1	7.9624732	4.5576577
127	DW	[10]	ore	osun	1	A557T	262	1	0	7.9624732	4.5576577
128	DX	[10]	ore	osun	1	A564T	262	1	0	7.9624732	4.5576577
129	DY	[10]	ore	osun	1	A569T	262	1	0	7.9624732	4.5576577
130	DZ	[10]	ore	osun	1	T573A	262	1	0	7.9624732	4.5576577
131	EA	[10]	ore	osun	1	A578T	262	1	0	7.9624732	4.5576577
132	EB	[10]	ore	osun	1	A578S	262	1	0	7.9624732	4.5576577
133	EC	[10]	ore	osun	1	A578S	262	1	0	7.9624732	4.5576577
134	ED	[10]	ore	osun	1	A578S	262	1	0	7.9624732	4.5576577
135	EE	[10]	ore	osun	1	A578S	262	1	0	7.9624732	4.5576577
136	EF	[10]	ore	osun	1	A578S	262	1	0	7.9624732	4.5576577
137	EG	[10]	ore	osun	1	C580Y	262	1	0	7.9624732	4.5576577
138	EH	[10]	ore	osun	1	V589V	262	1	1	7.9624732	4.5576577
139	EI	[10]	ore	osun	1	G595D	262	1	0	7.9624732	4.5576577
140	EJ	[10]	ore	osun	1	Q613H	262	1	0	7.9624732	4.5576577
141	EK	[10]	ore	osun	1	Q613H	262	1	0	7.9624732	4.5576577
142	EL	[10]	ore	osun	1	F614L	262	1	0	7.9624732	4.5576577

s/n	ID	Author	Location	State	N	SNPs	Sample size	Domain (441 to 726)	Syn /Non-syn	Lat	Lon
143	EM	[10]	ore	osun	1	S623N	262	1	0	7.9624732	4.5576577
144	EN	[10]	ore	osun	1	A626T	262	1	0	7.9624732	4.5576577
145	EO	[10]	ore	osun	1	A627T	262	1	0	7.9624732	4.5576577
146	EP	[10]	ore	osun	1	Y630Y	262	1	1	7.9624732	4.5576577
147	EQ	[10]	ore	osun	1	Y630Y	262	1	1	7.9624732	4.5576577
148	ER	[10]	ore	osun	1	G638G	262	1	1	7.9624732	4.5576577
149	ES	[10]	ore	osun	1	I646M	262	1	0	7.9624732	4.5576577
150	ET	[10]	ore	osun	1	Q652END	262	1	0	7.9624732	4.5576577
151	EU	[10]	ore	osun	1	Y653F	262	1	0	7.9624732	4.5576577
152	EV	[10]	ore	osun	1	P655S	262	1	0	7.9624732	4.5576577
153	EW	[10]	ore	osun	1	N657S	262	1	0	7.9624732	4.5576577
154	EX	[10]	ore	osun	1	Q661P	262	1	0	7.9624732	4.5576577
155	EY	[10]	ore	osun	1	E668K	262	1	0	7.9624732	4.5576577
156	EZ	[10]	ore	osun	1	E668Q	262	1	0	7.9624732	4.5576577
157	FA	[10]	ore	osun	1	E668E	262	1	1	7.9624732	4.5576577
158	FB	[10]	ore	osun	1	T677I	262	1	0	7.9624732	4.5576577
159	FC	[10]	ore	osun	1	G687G	262	1	1	7.9624732	4.5576577
160	FD	[10]	ore	osun	1	E688Q	262	1	0	7.9624732	4.5576577
161	FE	[10]	ore	osun	1	G690G	262	1	1	7.9624732	4.5576577
162	FF	[10]	ore	osun	1	V692I	262	1	0	7.9624732	4.5576577
163	FG	[10]	ore	osun	1	V692I	262	1	0	7.9624732	4.5576577
164	FH	[10]	ore	osun	1	V692I	262	1	0	7.9624732	4.5576577
165	FI	[10]	ore	osun	1	V692I	262	1	0	7.9624732	4.5576577
166	FJ	[10]	ore	osun	1	V692L	262	1	0	7.9624732	4.5576577
167	FK	[10]	ore	osun	1	F699F	262	1	1	7.9624732	4.5576577
168	FL	[10]	ore	osun	1	D702K	262	1	0	7.9624732	4.5576577

Table 3: Summary of imported *PfK13* polymorphisms studies from Nigeria reported in other countries

SNPs	Author	Country	No of SNPs	Sample size
S693F	[11]	Henan, China	1	157
Q613H	[11]	Henan, China	1	157
C469C	[11]	Henan, China	1	157
G496G	[11]	Henan, China	1	157
P441S	[12]	Zhejiang province, China	2	156
N458D	[12]	Zhejiang province, China	1	156
V650F	[12]	Zhejiang province, China	1	156
A676S	[12]	Zhejiang province, China	1	156
V589V	[12]	Zhejiang province, China	2	156
A578S	[13]	Shandong Province, China.	1	88
F442L	[13]	Shandong Province, China.	1	88
N664N	[13]	Shandong Province, China.	1	88
P443A	[13]	Shandong Province, China.	1	88
Q613H	[13]	Shandong Province, China.	3	88
E602D	[14]	Turkey	1	6
C469C	[15]	Shandong and Guangxi, China	1	144
A675V**	[16]	USA	1	56
C469C	[17]	China	1	152
G496G	[17]	China	1	152
A627A	[17]	China	1	152
C469C	[18]	Anhui, china	1	27
F446I*	[19]	Yunnan, China	1	72
Q469Q	[20]	New South Wales, Australia	1	28
A621A	[20]	New South Wales, Australia	1	28
G496G	[21]	Henan, China	1	72
K610R	[21]	Henan, China	1	72
A626T	[21]	Henan, China	1	72
A627A	[21]	Henan, China	1	72

References

[1] Mary Aigbiremo Oboh, Daouda Ndiaye, Hiasindh Ashmi Antony, Aida Sadikh Badiane, Upasana Shyamsunder Singh, Nazia Anwar Ali, Praveen Kumar Bharti, and Aparup Das. Status of artemisinin resistance in malaria parasite plasmodium falciparum from molecular analyses of the kelch13 gene in southwestern nigeria. *BioMed research international*, 2018(1):2305062, 2018.

[2] Fehintola V Ajogbasile, Paul E Oluniyi, Adeyemi T Kayode, Kazeem O Akano, Benjamin B Adegboyega, Courage Philip, Nnenna Ogbulafor, Henrietta U Okafor, Stephen Oguche, Robinson D Wammanda, et al. Molecular profiling of the artemisinin resistance kelch 13 gene in plasmodium falciparum from nigeria. *PLoS One*, 17(2):e0264548, 2022.

[3] Abel O Idowu, Wellington A Oyibo, Sanjib Bhattacharyya, Manjeet Khubbar, Udoma E Mendie, Violet V Bumah, Carolyn Black, Joseph Igietseme, and Anthony A Azenabor. Rare mutations in pfmdr1 gene of plasmodium falciparum detected in clinical isolates from patients treated with anti-malarial drug in nigeria. *Malaria journal*, 18(1):319, 2019.

[4] Moses Ikegbunam, Johnson A Ojo, Kossiwa Kokou, Ugonna Morikwe, Chukwuemeka Nworu, Chibuzo Uba, Charles Esimone, Thirumalaisamy P Velavan, and Olusola Ojurongbe. Absence of plasmodium

Table 4: Summary of sequencing methods used for the referenced studies

Reference No.	Sequencing Method
1	Sanger sequencing
2	Sanger sequencing
4	Sanger sequencing and Next Generation Sequencing (NGS)
5	Sanger sequencing
6	Sanger sequencing
7	Sanger sequencing
8	Not specified
9	Sanger sequencing
10	Sanger sequencing
11	Sanger sequencing
12	Sanger sequencing
13	Sanger sequencing
14	Sanger sequencing
15	Sanger sequencing
16	NGS
17	Sanger sequencing
18	Sanger sequencing
19	Sanger sequencing
20	Sanger sequencing
21	Sanger sequencing

falciparum artemisinin resistance gene mutations eleven years after the adoption of artemisinin-based combination therapy in nigeria. *Malaria Journal*, 20(1):434, 2021.

[5] Monday Tola, Olumide Ajibola, Emmanuel Taiwo Idowu, Olusesan Omidiji, Samson Taiwo Awolola, and Alfred Amambua-Ngwa. Molecular detection of drug resistant polymorphisms in plasmodium falciparum isolates from southwest, nigeria. *BMC research notes*, 13(1):497, 2020.

[6] Uche Igbasi, Wellington Oyibo, Sunday Omilabu, Hong Quan, Shen-Bo Chen, Hai-Mo Shen, Jun-Hu Chen, and Xiao-Nong Zhou. Kelch 13 propeller gene polymorphism among plasmodium falciparum isolates in lagos, nigeria: Molecular epidemiologic study. *Tropical Medicine & International Health*, 24(8):1011–1017, 2019.

[7] Joel Paul, Nelson J Nwankwo, M Simon, Shangshikmwa K Gaknung, Timloh Fakdul, Dinci T Davou, Mary M Mankilik, Richard J Kutshik, Bitrus Yakubu, and Ishaya Y Longdet. Non-synonymous mutations associated with plasmodium falciparum artemisinin resistant gene (pfkelch13) in malaria cases, jos nigeria. *Journal of Advances in Biology & Biotechnology*, 26(10):28–38, 2023.

[8] Olajide Joseph Afolabi, Olubanke Rosena Oluwafemi, and Mobolanle Oladipo Oniya. P fmdr 1 and kelch 13 genes distribution among children that are 5 years and below in akure, nigeria. *Journal of Parasitic Diseases*, 47(1):59–67, 2023.

[9] Umar F Abubakar, Ruqayya Adam, Muhammad M Mukhtar, Abdullahi Muhammad, Adamu A Yahuza, and Sulaiman S Ibrahim. Identification of mutations in antimalarial resistance gene kelch13 from plasmodium falciparum isolates in kano, nigeria. *Tropical medicine and infectious disease*, 5(2):85, 2020.

[10] Alexandra Martín Ramírez, Akeem Abiodun Akindele, Vicenta González Mora, Luz García, Nicole Lara, Eva de la Torre-Capitán Matías, Irene Molina de la Fuente, Sulaiman Adebayo Nassar, Thuy-Huong

Ta-Tang, Agustín Benito, et al. Mutational profile of pfdhfr, pfdhps, pfmdr1, pfcrt and pfk13 genes of p. falciparum associated with resistance to different antimalarial drugs in osun state, southwestern nigeria. *Tropical Medicine and Health*, 53(1):1–15, 2025.

[11] Dongyang Zhao, Hongwei Zhang, Penghui Ji, Suhua Li, Chengyun Yang, Ying Liu, Dan Qian, Yan Deng, Hao Wang, Deling Lu, et al. Surveillance of antimalarial drug-resistance genes in imported plasmodium falciparum isolates from nigeria in henan, china, 2012–2019. *Frontiers in cellular and infection microbiology*, 11:644576, 2021.

[12] Xiaoxiao Wang, Xuan Zhang, Hualiang Chen, Jiaqi Zhang, Qiaoyi Lu, Wei Ruan, and Feng Ling. Molecular epidemiology of drug resistance genes in plasmodium falciparum isolates imported from nigeria between 2016 and 2020: continued emergence of fully resistant pfdhfr-pfdhps alleles. *Microbiology Spectrum*, 10(5):e00528–22, 2022.

[13] Xiangli Kong, Jun Feng, Yan Xu, Ge Yan, and Shuisen Zhou. Molecular surveillance of artemisinin resistance-related pfk13 and pfcrt polymorphisms in imported plasmodium falciparum isolates reported in eastern china from 2015 to 2019. *Malaria Journal*, 21(1):369, 2022.

[14] Kübra Dilan Avcı, Mehmet Karakuş, and Kadriye Kart Yaşar. Molecular survey of pfmdr-1, pfcrt, and pfk13 gene mutations among patients returning from plasmodium falciparum endemic areas to turkey. *Malaria Journal*, 23(1):286, 2024.

[15] Jun Feng, Dongmei Xu, Xiangli Kong, Kangming Lin, He Yan, Xinyu Feng, Hong Tu, and Zhigui Xia. Characterization of pfmdr1, pfcrt, pfk13, pfubp1, and pfap2mu in travelers returning from africa with plasmodium falciparum infections reported in china from 2014 to 2018. *Antimicrobial Agents and Chemotherapy*, 65(7):10–1128, 2021.

[16] M Andreina Pacheco, Esha R Kadakia, Zainab Chaudhary, Douglas J Perkins, Julia Kelley, Shashidhar Ravishankar, Michael Cranfield, Eldin Talundzic, Venkatachalam Udhayakumar, and Ananias A Escalante. Evolution and genetic diversity of the k13 gene associated with artemisinin delayed parasite clearance in plasmodium falciparum. *Antimicrobial agents and chemotherapy*, 63(8):10–1128, 2019.

[17] Fang Huang, He Yan, Jing-Bo Xue, Yan-Wen Cui, Shui-Sen Zhou, Zhi-Gui Xia, Rabindra Abeyasinghe, Pascal Ringwald, and Xiao-Nong Zhou. Molecular surveillance of pfcrt, pfmdr1 and pfk13-propeller mutations in plasmodium falciparum isolates imported from africa to china. *Malaria journal*, 20(1):73, 2021.

[18] He Yan, Xiangli Kong, Tao Zhang, Huihui Xiao, Xinyu Feng, Hong Tu, and Jun Feng. Prevalence of plasmodium falciparum kelch 13 (pfk13) and ubiquitin-specific protease 1 (pfubp1) gene polymorphisms in returning travelers from africa reported in eastern china. *Antimicrobial Agents and Chemotherapy*, 64(11):10–1128, 2020.

[19] Yan He, Susana Campino, Ernest Diez Benavente, David C Warhurst, Khalid B Beshir, Inke Lubis, Ana Rita Gomes, Jun Feng, Wang Jiazhi, Xiaodong Sun, et al. Artemisinin resistance-associated markers in plasmodium falciparum parasites from the china-myanmar border: predicted structural stability of k13 propeller variants detected in a low-prevalence area. *PLoS One*, 14(3):e0213686, 2019.

[20] Christiane Prosser, Wieland Meyer, John Ellis, and Rogan Lee. Resistance screening and trend analysis of imported falciparum malaria in nsw, australia (2010 to 2016). *PLoS One*, 13(5):e0197369, 2018.

[21] Chengyun Yang, Hongwei Zhang, Ruimin Zhou, Dan Qian, Ying Liu, Yuling Zhao, Suhua Li, and Bianli Xu. Polymorphisms of plasmodium falciparum k13-propeller gene among migrant workers returning to henan province, china from africa. *BMC infectious diseases*, 17(1):560, 2017.
